# The relationship between stress and clinical high-risk symptoms of psychosis in daily life: impact of contemporaneous paths on cross-lagged effects

**DOI:** 10.1017/S0033291725000364

**Published:** 2025-03-03

**Authors:** Marialuisa Cavelti, Janko M. Kaeser, Silvano Sele, Thomas Berger, Michael Kaess, Jochen Kindler, Chantal Michel

**Affiliations:** 1 University Hospital of Child and Adolescent Psychiatry and Psychotherapy, University of Bern, Bern, Switzerland; 2 Department of Clinical Psychology and Psychotherapy, University of Bern, Bern, Switzerland; 3 Department of Child and Adolescent Psychiatry, Centre for Psychosocial Medicine, University of Heidelberg, Heidelberg, Germany

**Keywords:** at-risk mental state, ecological momentary assessment, experience sampling method, psychosis risk, residual dynamic structural equation modelingling, ultra-high risk

## Abstract

**Background:**

This study aimed to deepen the understanding of the psychological mechanisms underlying the formation and maintenance of clinical high-risk symptoms for psychosis (CHR-P) in real-life contexts. Specifically, it examined whether (i) momentary feelings of stress increase the frequency of CHR-P symptoms, or conversely, (ii) CHR-P symptoms increase the intensity of stress. Additionally, potential moderators of the relationship between stress and CHR-P symptoms were explored.

**Methods:**

Using Ecological Momentary Assessment, 79 patients (age: 11–36; 50.6% female) recruited from an early detection center for psychosis, reported their momentary stress levels and the frequency of CHR-P symptoms eight times a day for seven days. Time series data were analyzed using residual dynamic structural equation modeling in a random intercept cross-lagged panel design, comparing differently modeled contemporaneous effects.

**Results:**

There was no evidence of a contemporaneous or temporal link between stress on CHR-P symptoms. However, a contemporaneous effect of CHR-P symptoms on stress was found, while the corresponding temporal effect was not significant. The severity of interview-assessed CHR-P symptoms, age, and type of CHR-P symptoms (i.e., basic symptoms vs. [attenuated] positive symptoms) did not affect the contemporaneous effect of CHR-P symptoms on stress. However, nonperceptive symptoms had a greater contemporaneous effect on stress than perceptive symptoms.

**Conclusions:**

The findings suggest a greater contemporaneous impact of CHR-P symptoms on stress than vice versa. The experience of nonperceptive symptoms, in particular, may alter the appraisal of stress in daily life and represent a target for early interventions in real-time daily life (i.e., ecological momentary interventions).

## Introduction

Psychotic disorders, though relatively uncommon, are among the most disabling mental health conditions (GBD 2019 Mental Disorders Collaborators, 2022). They are typically preceded by clinical high-risk symptoms for psychosis (CHR-P); early signs of the disorder that may appear alongside other mental health issues (e.g., depressed mood or increased anxiety) and psychosocial difficulties, prompting individuals to seek help (Fusar-Poli et al., 2013). CHR-P symptoms are key targets for prevention and early intervention, aimed at alleviating early symptoms and distress, and potentially delaying or preventing progression to a full-blown psychotic or nonpsychotic disorder (Fusar-Poli et al., 2020; Shah et al., 2022).

Recent research has increasingly focused on psychological mechanisms involved in the development and persistence of psychotic experiences that can inform early intervention efforts (Myin-Germeys & van Os, 2007). Stress has long been acknowledged as an important factor in the etiology of psychosis. The vulnerability-stress model proposes that psychotic symptoms emerge when stressors exceed the individual’s vulnerability level (Nuechterlein et al., 1994). In line with this, critical life events (e.g., childhood adversity; Trotta et al., 2015), environmental stressors (e.g., urban life; Fett et al., 2019), and interpersonal stressors (e.g., bullying, high expressed emotions; Cunningham et al., 2016; Ma et al., 2021) have been linked to increased symptomatology (on the subclinical and clinical level) and increased relapse rates among people with psychotic disorders. However, retrospective assessments are prone to recall biases. Ecological momentary assessment (EMA) overcomes this by evaluating psychiatric symptoms and emotional responses in real time (Myin-Germeys et al., 2018). EMA studies have shown that daily life stressors (e.g., stressful activities, events, and social situations) are associated with increased psychotic responses in individuals across all levels of psychosis liability (including those with elevated familial risk, CHR-P symptoms, psychotic disorders, and transdiagnostic samples; Klippel et al., 2017, 2018; Monsonet et al., 2022; Paetzold et al., 2021; Radley et al., 2022; Rauschenberg et al., 2021; Reininghaus, Gayer-Anderson, et al., 2016; Reininghaus, Kempton, et al., 2016; Schick et al., 2023; Van Der Steen et al., 2017), supporting the idea of etiological continuity across the psychosis spectrum continuum (Klippel et al., 2017; Monsonet et al., 2022; Van Os & Linscott, 2012). Interestingly, recent findings suggest that the impact of daily life stress on psychotic experiences may be mediated by negative affect (Klippel et al., 2017, 2022; Kramer et al., 2014; Monsonet et al., 2022; Radley et al., 2022; Reininghaus, Kempton, et al., 2016), potentially indicating an affective pathway to psychosis (Myin-Germeys & van Os, 2007).

It is also plausible that psychotic experiences may precede distress rather than result from it, especially in individuals with CHR-P symptoms who often find these new, unusual experiences perplexing (Judge et al., 2008). This reverse pathway has received less research attention, and existing studies have produced inconsistent results. While one study identified an indirect temporal effect from psychotic experiences on stress, mediated by negative affect, across different adult samples along the psychosis spectrum (including CHR-P individuals; Monsonet et al., 2022), in two other studies, this indirect effect turned insignificant when using longitudinal modeling or taking covariates into account (Klippel et al., 2022; Radley et al., 2022).

Current EMA-based research on the link between momentary stress and psychotic experiences is limited. Most studies focus on either contemporaneous associations or the unidirectional pathway from stress to psychotic experiences, with the few studies exploring bidirectional relationships yielding inconsistent findings (Klippel et al., 2022; Monsonet et al., 2022). Examining bidirectional pathways is crucial, as neglecting these effects can lead to biased estimates in unidirectional models (Cole & Maxwell, 2003) and obscure potential complex interactions between etiological factors and symptoms. Further limitations include the lack of attention to contemporaneous effects between stress and psychotic symptoms within cross-lagged panel models (CLPM), which can bias cross-lagged estimates (Muthén & Asparouhov, 2024), and the insufficient differentiation between within-person processes and stable between-person differences, which can be best achieved by random intercept cross-lagged panel modeling (RI-CLPM; Hamaker et al., 2015; Lucas, 2023).

To address these gaps, this study examined the bidirectional relationships between EMA-measured stress and CHR-P symptoms using residual dynamic structural equation modeling (R-DSEM) within a RI-CLPM framework to simultaneously estimate contemporaneous and temporal effects. We hypothesized that (1) momentary stress increases the frequency of CHR-P symptoms and conversely (2) CHR-P symptoms intensify stress. Additionally, we explored whether these relationships are moderated by (3) the severity of interview-assessed CHR-P symptoms or (4) age at assessment. We also investigated, for the first time, differences (5) between two CHR-P criteria sets – attenuated (APS) or brief (limited) intermittent psychotic symptoms (B[L]IPS), the two symptomatic ultra-high risk (UHR) criteria, versus basic symptoms (Fusar-Poli et al., 2013; Schultze-Lutter et al., 2015) – as well as (6) between perceptive CHR-P and nonperceptive (cognitive) CHR-P symptoms (Cornblatt et al., 2015; Michel, Lerch, et al., 2022; Schimmelmann et al., 2015; Schultze-Lutter et al., 2020). This approach was driven by evidence highlighting the clinical relevance of these distinctions: APS/B(L)IPS is associated with an imminent risk of psychosis, while BS can be detected earlier in the course of psychotic development (Fusar-Poli et al., 2013; Schultze-Lutter et al., 2015). Additionally, in young people, perceptive symptoms (e.g., visual or acoustic perceptive disturbances or [attenuated] hallucinations), though more frequent, tend to be less stable and less clinically significant, showing weaker associations with functional deficits and the presence of mental disorders compared to nonperceptive symptoms (e.g., derealization or [attenuated] delusional ideas; Cornblatt et al., 2015; Michel, Lerch, et al., 2022; Schimmelmann et al., 2015; Schultze-Lutter et al., 2020). For a more detailed summary of the relevant literature regarding the two complementary criteria sets defining the CHR-P state (i.e., UHR and BS), we refer to page 3 in Supplementary Materials (SM).

## Methods

### Participants and procedures

Participants (*N* = 80) were recruited from the ‘Bern Early Recognition and Intervention Centre’ (FETZ Bern; Michel, Kaess, et al., 2022). The FETZ Bern is a state-of-the art psychosis-risk detection center for patients between 8 and 40 years of age with putative psychotic symptoms, which offers a comprehensive diagnostic assessment of CHR-P and psychotic disorders according to international gold standards (Schultze-Lutter et al., 2015). Patients of the catchment area of the canton Bern (~1.5 million inhabitants) can either be admitted to the FETZ Bern by physicians and psychosocial institutions if there is suspicion of early psychotic development or enroll on their own initiative. Exclusion criteria include (1) a psychotic disorder diagnosis according to DSM-IV and ICD-10 in the past, (2) a diagnosis of dementia, delirium, amnesia, or other neurological disorder, and (3) general medical conditions known to impact the central nervous system (Michel, Kaess, et al., 2022). The current consecutive sample encompasses attendees of the time period from January 2019 to October 2021. It partly overlaps with the sample of the study by Michel, Lerch, et al. (2022).

EMA data originate from a study on the “Exploratory behavioural and biological investigation of psychosis risk symptoms in children, adolescents and adults”. Eligibility for the study required that patients met the entry criteria for the service (described above) and provided informed consent. For minors, informed consent of the parents with assent of the child was obtained. The ethics committee of the Canton Bern gave approval for all procedures (ID PB_2016-01,991, ID 2018–00,951), which comply with the ethical standards of the Helsinki Declaration.

## Measures

### Interview-based assessment of CHR-P symptoms

Well-established semistructured interviews were used to assess CHR-P symptoms and criteria (Fux et al., 2013; McGlashan et al., 2010; Schultze-Lutter et al., 2007). APS/B(L)IPS symptoms and criteria were assessed with the Structured Interview for Psychosis-Risk Syndromes (SIPS; McGlashan et al., 2010), which has demonstrated excellent overall psychometric properties, including robust validity and high interrater reliability (median kappa = 0.89; Shapiro et al., 2019; Woods et al., 2019). It measures five attenuated positive psychotic symptoms (i.e., delusions, paranoia, grandiosity, hallucinations, and disorganized speech). They are rated on a scale from 0 (not present) to 6 (severe and psychotic). For analysis, a total APS/B(L)IPS severity score was created, ranging from 0 to 5, as following: First, the five symptom ratings were dichotomized, with ratings 0 to 2 scored as ‘0’ (absent) and ratings 3 to 6 scored as ‘1’ (present). Next, the five dichotomized symptom scores were summed up. BS were assessed using the Schizophrenia Proneness Instrument (SPI-A for adults, Schultze-Lutter et al., 2007; SPI-CY for children and adolescents, Fux et al., 2013), which has demonstrated good-to-excellent discriminative validity and good interrater reliability, with concordance rates reaching 89% (Fux et al., 2013; Chantal Michel et al., 2014; Frauke Schultze-Lutter et al., 2007). The SPI-A/-CY assess 14 cognitive and perceptive BS that are rated on a severity scale according to their maximum frequency of their occurrence within the past three months, ranging from 0 (absent) to 6 (extreme). Symptoms may also be rated as 7 (basic symptom has always been present in the same severity, trait), 8 (basic symptom is definitely present, but its frequency of occurrence is unknown), and 9 (basic symptom can neither be definitively confirmed nor ruled out). A BS severity score was created in a similar fashion as the APS/B(L)IPS severity score: First, the BS ratings were dichotomized, with the ratings 0, 7, and 9 scored as ‘0’ (absent) and ratings 1 to 6, and 8 scored as ‘1’ (present). Then, the dichotomized scores were summed up creating a BS severity score ranging from 0 to 14 used in the analyses.

### EMA

Participants received a smartphone with the movisensXS experience sampling application (Movisens GmbH, Karlsruhe, Germany), which served as an electronic diary. After instructions in the use of the application, a 28-item EMA survey was delivered over a 7-day period, with 8 prompts per day, pseudo-randomly distributed between 8 am and 10 pm and with a minimum of 25 min between prompts. To enhance compliance, participants could postpone each prompt once for 5, 10, or 15 min. At each EMA prompt, participants were asked to rate one item on their subjective stress level (‘How stressed are you feeling right now?’) as well as the frequency of the occurrence of 14 BS and seven APS/B(L)IPS since the last beep. Item formulation for CHR-P symptoms was based on SPI-A/SPI-CY (Fux et al., 2013; Schultze-Lutter et al., 2007) as well as Appendix B of the SIPS (McGlashan et al., 2010). For analysis, we calculated for each EMA prompt (1) the mean score over all 21 CHR-P symptoms (mCHR); (2) separate mean scores for BS and APS/B(L)IPS; and (3) separate mean scores for perceptive (PERC) and nonperceptive (NONP) CHR-P symptoms. A more detailed description of the EMA item development, the EMA sampling scheme, and the psychometric properties of the EMA measures can be found in the SM and in Michel, Lerch, et al. (2022).

### Statistical analyses

Using R-DSEM, we chose a latent multilevel modeling approach to investigate the processes in the given time series, including autoregressive effects. This method is particularly well suited for handling missing data (Asparouhov et al., 2018; Asparouhov & Muthén, 2020), a common problem in EMA studies (Myin-Germeys et al., 2018). By reducing data requirements per participant for model fitting, it minimizes the exclusion of participants with incomplete data, which can introduce biases to the results.

Initially, we fitted three basic models where the autoregression of stress and mCHR, and the cross-lagged effects between the two variables was analyzed with a lag of 1. Due to the semirandom nature of EMA prompts and the presence of missing data, the measurement intervals varied among participants, leading to misaligned data. To achieve temporal synchronization across participants, a uniform time grid was implemented with 24 measurement intervals per day (i.e., one per hour). Hours without measurement data were accounted for by inserting missing values using the ‘TINTERVAL’ command in Mplus (Asparouhov & Muthén, 2020). This approach allowed R-DSEM to estimate a 1-h lag between measurement points. For measurements occurring further apart (e.g., overnight or due to missing data resulting from low adherence), the model accounts for a decay of effects over times, reflected by lower autocorrelations between distant measurement points (Asparouhov et al., 2018; McNeish & Hamaker, 2020).

**Figure 1.**
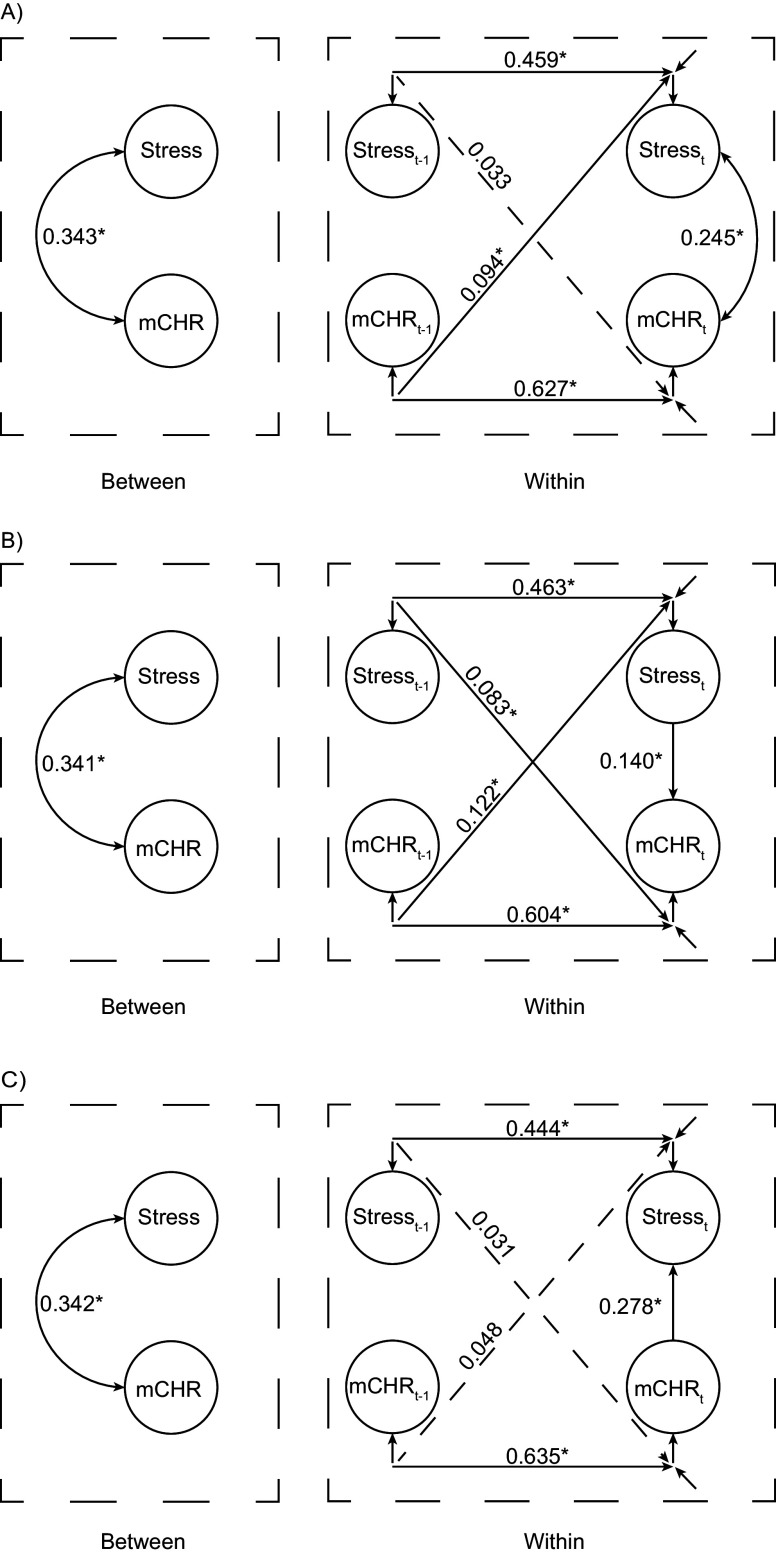
Random intercept cross-lagged panel models with varying lag0-effect. *N* = 79. Number of Observations: 3’063. Depiction of the three basic model structures, including the autocorrelative, cross-lagged, and contemporaneous effects of stress and the mean score over all CHR-P symptoms (mCHR). Dashed lines represent insignificant paths. In Model A, the lag0-effect is modeled as a covariation without a fixed directionality. B shows a directed lag0-effect of stress on mCHR. In Model C, the opposite directed lag0-effect of mCHR on stress is shown. See Table 3 for detailed results.

The difference between the three basic models was the way in which the contemporaneous relationship (i.e., lag0-effect) between stress and mCHR was modeled (1) as a covariance (see Model A in Figure 1); (2) as a directed lag0-effect of stress on mCHR (see Model B); and (3) as a directed lag0-effect of mCHR on stress (see Model C). Modeling directed lag0-effects is beneficial in time series, as it approximates lags that are shorter than the defined time interval between two consecutive measurements but greater than zero (Epskamp et al., 2018; Muthén & Asparouhov, 2024). Muthén and Asparouhov (2024) recommend to consider the results from all possibilities of modeling the contemporaneous effect when interpreting cross-lagged effects (i.e., lag1-effects). Accordingly, we compared the three models outlined above and selected the best fitting model based on the deviance information criterion (DIC; e.g., Meyer, 2016) and path structure, considering parsimony and plausibility according to the Occam’s Razor principle (Domingos, 1999). Finally, random effects for significant paths and random residual variances for stress and mCHR were included into the best fitting model to account for within-subject variation, thereby addressing intraindividual heterogeneity often present in individual time series data. The minimum number of iterations before convergence was set at 2000. A potential scale reduction (PSR) value close to 1 was considered a sign of successful convergence, as recommended by Asparouhov and Muthén (2010).

To examine the between-level moderators such as age, sex, and interview-assessed severity of BS and APS/B(L)IPS, we regressed these variables on the random effects of the significant paths in the final model. The Wald test was applied to jointly assess the impact of the moderators on relevant paths in the final model (Molenberghs & Verbeke, 2007).

Moreover, to explore whether momentary stress had differential associations with EMA-based APS/B(L)IPS and BS, the autocorrelations of stress, APS/B(L)IPS, and BS, and the cross-lagged (lag1-) effects and contemporaneous (lag0-) effects between stress, APS/B(L)IPS, and BS were simultaneously included into the model. The same procedure was applied to investigate differential associations of stress with nonperceptive (NONP) and perceptive (PERC) symptoms.

Data were prepared and analyzed descriptively with R (R Core Team, 2023). R-DSEM was conducted using Mplus (Muthén & Muthén, 2017). For Bayesian estimation, we used the default prior by Mplus for all model parameters. Mplus results were processed using the R package *MplusAutomation* (Hallquist & Wiley, 2018). The code for the R-DSEM models is provided in the SM.

## Results

### Sample characteristics

Overall, 80 patients enrolled in the EMA phase, of which one person completed only one single survey during the entire EMA phase and was therefore not included in further analyses due to a lack of variance. Concerning diagnostic results, 45 participants were diagnosed with a current CHR-P state, and 7 participants with a first-episode psychotic disorder. Twenty-six participants fulfilled neither criteria for a CHR-P state nor a psychotic disorder, and one participant did not complete the diagnostic assessment. Due to missing data, this participant had to be excluded from the moderator analysis examining the effect of interview-based APS/B(L)IPS and BS severity on the associations between stress and mCHR. Further sociodemographic and clinical characteristics are shown in Table 1.

**Table 1.** Sociodemographic and clinical characteristics

	*M* (SD)/*n* (%)	
Age (years)	18.99	(4.93)
Sex		
Female	40	50.6
Male	39	49.4
Highest level of education^a^		
Early childhood education (ISCED level 0)	4	5.1
Primary education (ISCED 1)	2	2.5
Lower secondary education (ISCED 2)	28	35.4
Upper secondary education (ISCED 3)	35	44.3
Tertiary education (ISCED 5)	7	8.9
Not applicable	1	1.3
Other	1	1.3
Not specified	1	1.3
Mental, behavioral and neurodevelopmental disorders^b^		
F10–F19	12	15.2
F20–F29	7	8.9
F30–F39	52	65.8
F40–F48	31	39.2
F50–F59	3	3.8
F60–F69	3	3.8
F80–F89	2	2.5
F90–F99	6	7.6
CHR-P state		
Present	45	57.0
Not present; no F2 diagnosis	26	32.9
Not applicable; with F2 diagnosis	7	8.9
No data available	1	1.3
Severity of APS/B(L)IPS	2.01	(1.15)
Severity of BS	3.30	(2.58)

*Note*: *N* = 79. *M*, mean; *SD*, standard deviation; CHR-P, clinical high risk for psychosis; APS, attenuated psychotic symptoms; B(L)IPS, brief (limited) intermittent psychotic symptoms; BS, basic symptoms. A detailed description of the age range can be found in the SM (p. 16).

a Education levels are based on ISCED.

b No F0 or F7 disorders were diagnosed.

EMA adherence of the final sample (*N* = 79) was on average 69.24% (SD = 24.65%, range: 10.71–100%). Detailed information on EMA adherence is provided on page 8 of the SM. Descriptive statistics of the EMA variables are presented in Table 2, demonstrating significant intraindividual variation in the frequency of CHR-P symptoms across participants (see also Figure 2 on p. 10 in the SM).

**Table 2.** Mean and standard deviation (SD) of different individual summary statistics of the EMA data

Score	iMean	iMedian	iSD	iSkewness	iRMSSD
mCHR	22.88 (19.15)	21.79 (20.23)	8.22 (5.25)	0.80 (1.19)	8.68 (5.57)
Stress	38.75 (22.74)	37.26 (27.96)	19.29 (7.74)	0.50 (1.06)	22.28 (9.21)
APS/B(L)IPS	22.11 (20.90)	21.10 (22.27)	8.93 (5.48)	0.93 (1.37)	10.20 (6.42)
BS	23.26 (18.97)	22.19 (20.09)	8.99 (5.52)	0.78 (1.26)	9.57 (6.05)
Perc	17.68 (21.45)	16.41 (22.13)	7.57 (6.05)	1.30 (1.45)	8.93 (7.44)
Non-perc	24.50 (19.71)	23.41 (21.26)	9.24 (5.58)	0.79 (1.23)	9.73 (5.88)

*Note*: *N* = 79. The scores preceded by an ‘i’ are the averages of the individual-level statistics. For illustration, the iMedian consists of the mean over the median formed for each individual over the EMA time series. mCHR, mean over CHR-P symptoms; APS, attenuated psychotic symptoms; B(L)IPS, brief (limited) intermittent psychotic symptoms; BS, basic symptoms; Perc, perceptive symptoms; Non-perc, nonperceptive symptoms; RMSSD, root mean sum of squared distance; standard deviation in parentheses.

### Basic models

All models successfully converged at the predefined minimum of 2000 iterations. The PSR value was close to 1 for all models, signaling successful convergence. Table 3 presents the complete results of the basic Models A, B, and C, and Figure 1 provides a graphical representation of the paths. All three basic models showed a similarly strong correlation between stress and mCHR on the between level (standardized estimates [st.est]: 0.341–0.343) and similarly strong autocorrelative effects for stress and mCHR, respectively, (stress_st.est_: 0.444–0.463; mCHR_st.est_: 0.604–0.635) on the within level. In all three models, the differently modeled lag0-effect between stress and mCHR was significant (Model A_st.est_: 0.245, Model B_st.est_: 0.140, Model C_st.est_: 0.278).

**Table 3.** Results of the three simple RDSEM-models

Model A					
*Within level*	Estimate	SD	*P* ^c^	LCI	UCI
**Effects**					
Stress (T−1) → Stress (T)	0.459*	0.022	0.000	0.416	0.500
mCHR (T−1) → Stress (T)	0.094*	0.019	0.000	0.057	0.133
mCHR (T−1) → mCHR (T)	0.627*	0.018	0.000	0.590	0.661
Stress (T−1) → mCHR (T)	0.033	0.018	0.031	–0.002	0.069
Stress (T) ←→ mCHR (T)	0.245*	0.019	0.000	0.208	0.282
**Residual variances**					
Stress	0.751*	0.020	0.000	0.712	0.790
mCHR	0.592*	0.021	0.000	0.552	0.632
***R*** ^**2**^					
Stress	0.249*	0.020	0.000	0.210	0.288
mCHR	0.408*	0.021	0.000	0.368	0.448

*Between level*	Estimate	SD	*P* ^c^	LCI	UCI
**Effects**					
Stress ←→ mCHR	0.343*	0.108	0.002	0.113	0.536
Means					
Stress	1.705*	0.186	0.000	1.364	2.089
mCHR	1.178*	0.148	0.000	0.894	1.470
**Residual variances**					
Stress	1.000	0.000	-	-	-
mCHR	1.000	0.000	-	-	-
*Iterations* ^a^	2000				
*PSR* ^b^	1.011				
*DIC*	210506.807				

Model B					
*Within level*	Estimate	SD	*P* ^c^	LCI	UCI
**Effects**					
Stress (T−1) → Stress (T)	0.463*	0.025	0.000	0.413	0.511
mCHR (T−1) → Stress (T)	0.122*	0.026	0.000	0.073	0.173
mCHR (T−1) → mCHR (T)	0.604*	0.018	0.000	0.567	0.641
Stress (T−1) → mCHR (T)	0.083*	0.021	0.000	0.041	0.125
Stress (T) → mCHR (T)	0.140*	0.052	0.005	0.035	0.238
**Residual variances**					
Stress	0.752*	0.021	0.000	0.709	0.791
mCHR	0.572*	0.023	0.000	0.527	0.617
***R*** ^**2**^					
Stress	0.248*	0.021	0.000	0.209	0.291
mCHR	0.428*	0.023	0.000	0.382	0.473

*Between level*	Estimate	SD	*P* ^c^	LCI	UCI
**Effects**					
Stress ←→ mCHR	0.341*	0.108	0.002	0.124	0.540
Means					
Stress	1.713*	0.187	0.000	1.349	2.082
mCHR	1.179*	0.152	0.000	0.887	1.473
**Residual variances**					
Stress	1.000	0.000	-	-	-
PE	1.000	0.000	-	-	-
*Iterations* ^a^	2000				
*PSR* ^b^	1.010				
*DIC*	210790.777				

Model C					
*Within level*	Estimate	SD	*P* c	LCI	UCI
**Effects**					
Stress (T−1) → Stress (T)	0.444*	0.023	0.000	0.398	0.489
mCHR (T−1) → Stress (T)	0.048	0.043	0.146	−0.038	0.128
mCHR (T−1) → mCHR (T)	0.635*	0.017	0.000	0.602	0.668
Stress (T−1) → mCHR (T)	0.031	0.021	0.056	−0.009	0.074
mCHR (T) → Stress (T)	0.278*	0.073	0.000	0.131	0.409
**Residual variances**					
Stress	0.708*	0.027	0.000	0.655	0.761
mCHR	0.593*	0.020	0.000	0.551	0.631
***R*** ^**2**^					
Stress	0.292*	0.027	0.000	0.239	0.345
mCHR	0.407*	0.020	0.000	0.369	0.449

*Between level*	Estimate	SD	*P* c	LCI	UCI
**Effects**					
Stress ←→ mCHR	0.342*	0.108	0.002	0.124	0.542
Means					
Stress	1.714*	0.187	0.000	1.350	2.077
mCHR	1.179*	0.152	0.000	0.887	1.474
**Residual variances**					
Stress	1.000	0.000	-	-	-
mCHR	1.000	0.000			
*Iterations* ^a^	2000				
*PSR* ^b^	1.006				
*DIC*	210736.924				

*Notes. N* = 79. Number of observations = 3’063. All parameters in this table are standardized. mCHR, mean score over all CHR-P symptoms. P-value is two-tailed; LCI; lower part of confidence interval; UCI, upper part of confidence interval; DIC, Deviance Information Criterion; PSR, Potential Scale Reduction; ‘A → B’ symbolizes a directed effect of A on B (i.e., B regressed on A); ‘A ←→ B’ symbolizes an undirected covariation between A and B. ‘*’ = significant estimate.

a The minimum number of iterations was set at 2000. All three models converged at this minimum.

b Value of the last iteration.

c Values below 0.025 are considered significant.

Meaningful differences between the models emerged with regard to the cross-lagged effects: While in Model B, both cross-lagged paths showed significant but small effects (mCHR_*t*−1_ on stress*_t_* [st.est]: 0.122; stress_*t*−1_ on mCHR*_t_* [st.est]: 0.083), in Model A, only the cross-lagged path from mCHR_*t*−1_ to stress*_t_* showed a small significant effect (st.est: 0.094). In contrast, in Model C, none of the cross-lagged paths was statistically significant.

When evaluating model fit based on the global fit index DIC, Model A (DIC = 210506.807) was favored over Model C (DIC = 210736.924) and Model B (DIC = 210790.777). However, based on a Monte Carlo simulation, the DIC seems only reasonably comparable between Model C and Model B (see the SM for results and code), questioning the superiority of Model A. Considering the parsimony criterion, Model C explains the data with the fewest paths, followed by Model A, and finally, Model B. Considering both, model fit and parsimony, Model B exhibits the poorest model fit and uses the most paths to explain the data, favoring Models A and C over Model B. Neither Model A nor Model C shows a cross-lagged effect between stress_*t*−1_ and subsequent mCHR_t_, suggesting that, in Model B, the R-DSEM algorithm compensates for the restricted directed lag0-effect of stress*_t_* on mCHR*_t_* by employing the cross-lagged effect of stress_*t*−1_ on mCHR*_t_.* The results of the aforementioned Monte Carlo simulation support this conclusion (see SM).

Considering plausibility of paths, it is notable that Model C renders the cross-lagged path of mCHR_*t*−1_ on stress_t_ insignificant, which is significant in Model A (and B), while having the lag0-effect of the highest magnitude among the three models. Of note, the frequency of CHR-P symptoms is assessed from the time of the last beep, meaning the directed lag0-effect from mCHR_*t*_ on stress_t_ in Model C inherently includes a temporal order. In addition, as a directed lag0-effect approximates a model with a lag smaller than the defined time interval but greater than zero (Muthén & Asparouhov, 2024), Model C implies a link between preceding CHR-P symptoms and subsequent subjective stress, similar to the cross-lagged effect of Model A from mCHR_*t*−1_ on stress*_t_*, but within a smaller lag than the modeled one-hour time interval. Consequently, because Model C incorporates a directed effect of mCHR on stress similar to Model A but was more parsimonious, it was selected as the final model for further analysis.

Finally, we added random slopes to the significant paths in Model C (i.e., the autoregressive effects [stress_*t*−1_ on stress*_t_*, mCHR_*t*−1_ on mCHR*_t_*] and the directed lag0-effect of mCHR*_t_* on stress*_t_*) and a random residual variance for stress and mCHR. The comparison of Model C, with and without additional random effects, revealed no changes in the significance or sizes of the model paths (see SM, Table 6, for a full report).

### Moderating effects of age, sex, and interview-based CHR-P severity

The Wald test jointly examining the impact of age, sex, and interview-based CHR-P severity on the random slope of the lag0-effect from mCHR*_t_* on Stress_*t*_ was not statistically significant (*p* = 0.351), indicating that the moderators did not significantly influence the contemporaneous effect of psychotic symptoms on feelings of stress. See SM, Table 7, for a full report of the moderation model.

### Differential effects for APS/B(L)IPS versus BS

The directed lag0-effects of APS/B(L)IPS*_t_* on stress*_t_* (st.est: 0.156) and BS*_t_* on stress*_t_* (st.est: 0.136) were both significant and of similar magnitude (i.e., overlapping CIs). See SM, Table 8, for a full report of the model with CHR-P symptoms split into APS/B(L)IPS and BS.

### Differential effects for perceptive versus nonperceptive CHR-P symptoms

The directed lag0-effects of PERC*_t_* on stress*_t_* (st.est: 0.062) and NONP*_t_* on stress*_t_* (st.est: 0.196) were both significant, with the latter being of significantly greater magnitude (i.e., CIs not overlapping). See SM, Table 9 for a full report of the model with CHR-P symptoms split into perceptive versus nonperceptive symptoms.

## Discussion

### Principle findings

This study examined the bidirectional relationships between EMA-measured momentary feelings of stress and CHR-P symptoms using R-DSEM within a RI-CLPM design. We found no evidence for either a contemporaneous (i.e., lag0-) or a cross-lagged (i.e., lag1-) effect of stress on CHR-P symptoms. In contrast, there was a significant contemporaneous effect of CHR-P symptoms on stress, though the corresponding cross-lagged effect was not significant. The contemporaneous effect of CHR-P symptoms on stress was unaffected by the severity of interview-assessed CHR-P symptoms, age, and the differentiation between APS/B(L)IPS versus BS. However, nonperceptive symptoms had a stronger contemporaneous impact on stress than perceptive symptoms.

### Comparison with previous research

The absence of both contemporaneous (lag0-) and cross-lagged (lag1-) effects of stress on CHR-P symptoms in the current study contradicts previous reports of cross-sectional (Klippel et al., 2017, 2022; Rauschenberg et al., 2021; Reininghaus, Kempton, et al., 2016; Schick et al., 2023; Van Der Steen et al., 2017) and longitudinal effects (Monsonet et al., 2022; Paetzold et al., 2021; Radley et al., 2022; Schick et al., 2023) of momentary stress on psychotic symptoms in individuals across the psychosis severity spectrum (including CHR-P samples). It partially aligns with the study by Klippel et al. (2022) who reported that the cross-sectional effect of stress on psychotic experiences in patients with psychotic disorders, unaffected relatives, and healthy controls disappeared in the longitudinal models.

Few studies have examined the reverse pathway from psychotic experiences to stress. The contemporaneous (lag0-) effect of CHR-P symptoms on stress observed in our study is consistent with the cross-sectional findings reported by Klippel et al. (2022). Notably, similar to our findings, the directed effect of psychotic experiences on stress in Klippel et al.’s (2022) study disappeared in longitudinal models. Interestingly, they found that the magnitude of the cross-sectional pathway from psychotic experiences to momentary stress was significantly larger than the reverse pathway. Our finding of a contemporaneous (lag0-) effect of CHR-P symptoms on stress also aligns with Monsonet et al.’s (2022) findings on a temporal effect of psychotic-like experiences and paranoia on stress in individuals with high schizotypy, CHR-P, or first-episode psychosis. However, Monsonet et al. (2022) also found evidence supporting the reverse pathway, indicating a bidirectional relationship.

The discrepancies from previous findings may be attributed to methodological differences. Many existent studies have focused solely on the unidirectional pathway from stress to psychotic experiences, potentially leading to biased estimates (Cole & Maxwell, 2003), overlooked the influence of contemporaneous (lag0-) effects on cross-lagged estimates (Muthén & Asparouhov, 2024), or failed to adequately differentiate between within-person and between-person variations (Hamaker et al., 2015; Lucas, 2023). Additionally, unlike most previous EMA studies that assessed stress in relation to specific events or social stressors, this study measured feelings of stress independently. Moreover, our sample, with a mean age of 18.99 years (SD = 4.93), was substantially younger than the average age of participants in previous EMA studies in psychosis (*M* = 36.90, SD = 10.27 years), as reported in a recent meta-analysis (Bell et al., 2023). Finally, the time interval between assessments, which differs between the current and previous studies, can significantly impact findings on temporal effects. In this study, assessments were conducted eight times a day in a 14-h time window. Since CHR-P symptoms were assessed from the time of the last beep, a common approach in EMA research on psychotic symptoms (Bell et al., 2023), the actual time between the occurrence of CHR-P symptoms and the assessment of stress could potentially be as long as the time since the penultimate completed assessment (i.e., several hours). The observed directed contemporaneous (lag0)-effect of CHR-P symptoms on stress, without a corresponding crossed-lagged effect, suggests that the effect may occur within a timeframe greater than zero but shorten than the interval modeled in this study, which was 1 h (Epskamp et al., 2018; Muthén & Asparouhov, 2024). In EMA research, it is crucial that the assessment schemes match the effect of interest and its expected evolution. Since the timeframe in which the relationship between CHR-P symptoms and stress manifests is unknown, future EMA studies should explore the bidirectional relationship over time by systematically varying the intervals between assessments (Klippel et al., 2022; Reininghaus, Depp, & Myin-Germeys, 2016).

Our study expands on previous research by demonstrating that the directed contemporaneous (lag0)-effect of CHR-P symptoms on stress applies not only to APS/B(L)IPS, which indicate a more immediate risk for developing a psychotic disorder, but also to the more subtle BS that occur earlier in the prodromal phase (Fusar-Poli et al., 2013; Schultze-Lutter et al., 2015). Furthermore, the finding that the directed contemporaneous (lag0)-effect from CHR-P symptoms to stress is more pronounced for nonperceptive symptoms than for perceptive symptoms suggests that nonperceptive symptoms (i.e., thought interference, blockages, and perseveration, difficulties of discriminating between ideas and perceptions, captivation of attention by details of the visual field, inability to divide attention, derealization or [attenuated] delusional ideas) may be experienced as particularly stressful in daily life. This adds to previous research indicating that while perceptive symptoms are more frequent in young people with CHR-P, they appear to be less clinically relevant (Michel, Lerch, et al., 2022; Schimmelmann et al., 2015; Schultze-Lutter et al., 2020).

### Methodological considerations

The current findings should be considered in the light of methodological limitations. First, while the model selection process was guided by the DIC, as well as parsimony and plausibility of paths, the decision between Model A and Model C remains somewhat arbitrary. We cannot entirely rule out the possibility that Model A is the ‘true’ model. However, even if Model A is indeed the ‘true’ model, it would lead to a similar conclusion as Model C: there is a directed effect of CHR-P on stress, with no evidence supporting the reverse pathway. Second, while the current finding of a contemporaneous, directed effect of CHR-P symptoms on stress could be an indicator of causality (Granger, 1969), we cannot rule out the possibility that the link between CHR-P symptoms and stress arose due to third variables not considered in the current analysis. Third, additional psychological mechanisms not explored in the current study, such as aberrant salience, threat anticipation, negative affect, and self-esteem, may contribute to heightened psychotic experiences in real life (Monsonet et al., 2022; Reininghaus, Kempton, et al., 2016). These mechanisms warrant further investigation in future research. Fourth, EMA items were always presented in the same order, potentially introducing systematic biases due to the influence of earlier questions on *subsequent* ones. Fifth, since psychotic symptoms are infrequent phenomena, the EMA questions were designed to assess the occurrence of CHR-P symptoms between the current and the previous prompt (i.e., ‘since the last beep’). This approach is consistent with common practices in EMA research on psychotic symptoms (Bogudzińska et al., 2024). However, as CHR-P symptoms may have occurred before the actual prompt, the directed lag0-effect from CHR-P symptoms has a temporal component, representing a cumulative measure of CHR-P events occurring between two prompts. Accordingly, our EMA item formulation for CHR-P symptoms may have reduced the likelihood of a lag0-effect of stress*_t_* on CHR-P*_t_*, while it may have simultaneously increased the probability of a lag1-effect of stress_*t*−1_ on CHR-P*_t_.* Finally, EMA measures are based on participants’ subjective reports and may therefore be less reliable than interview assessments, as participants might interpret the questions differently. However, in a recent study with a sample largely overlapping with the current one, we found significant associations between interview- and EMA-based ratings of CHR-P symptoms, suggesting that EMA can reliably assess CHR-P symptoms (Michel, Lerch, et al., 2022). This is further supported by recent reviews demonstrating that EMA is a feasible, reliable, and valid assessment method in psychosis studies, including CHR-P research (Bell et al., 2023; Bogudzińska et al., 2024).

## Conclusion

Our findings, in the context of previous research, tentatively suggest that CHR-P symptoms exert a greater influence on stress than stress does on CHR-P symptoms. This effect is evident for both BS and APS/B(L)IPS and is more pronounced for nonperceptive symptoms compared to perceptive ones. In terms of theoretical implications, our results did not provide evidence supporting the idea that stress acts as a trigger for CHR-P symptoms, as proposed by the vulnerability-stress model (Nuechterlein et al., 1994). However, this should not be taken as a refutation of the model, which primarily addresses interindividual processes (e.g., suggesting that stress is associated with psychotic experiences at the group level) without specifying how this relationship unfolds at the intraindividual level. More specifically, the model does not clarify the specific timeframe within which the stress-psychosis relationship manifests in daily life. In addition, it has been developed with a primary focus on adults. This is a critical limitation, given that the majority of mental disorders typically emerge between the ages of 12 and 25, a period marked by significant neurobiological and psychosocial changes that increase vulnerability to mental health conditions. Addressing these developmental aspects is particularly urgent, as the declining mental health of young people in recent decades underscores the importance of refining models and interventions to mitigate the growing societal and individual impacts of untreated mental illness during this critical life stage (McGorry et al., 2024; Uhlhaas et al., 2023). Ultimately, a more formalized theory of stress reactivity in psychosis is needed to generate predictions about the empirical phenomena that should be observable (on the within- and between-person level) if the theory holds true (Borsboom & Haslbeck, 2024), and that also takes developmental aspects (e.g., age-related stressors, neurobiological maturation) more strongly into account. Regarding clinical implications, the findings may indicate that the occurrence of CHR-P symptoms influences stress appraisal in daily life, potentially through increased negative affect, such as anxiety (Klippel et al., 2022; Monsonet et al., 2022). Ecological momentary interventions could be employed to monitor CHR-P symptoms (especially nonperceptive symptoms) and stress, and provide real-time, personalized interventions to mitigate psychotic experiences and stress in everyday situations.

## Supporting information

Cavelti et al. supplementary material Cavelti et al. supplementary material

## Data Availability

Can be requested from the corresponding author. Code for R-DSEM models is provided in the SM. Additional code can be requested from the corresponding author.

## Supplementary material

The supplementary material for this article can be found at http://doi.org/10.1017/S0033291725000364.

## References

[r1] Asparouhov, T., Hamaker, E. L., & Muthén, B. (2018). Dynamic structural equation models. Structural Equation Modeling: A Multidisciplinary Journal, 25(3), 359–388. 10.1080/10705511.2017.1406803

[r2] Asparouhov, T., & Muthén, B. (2010). Bayesian analysis using Mplus: Technical implementation (Technical report, Version 3). College Station, TX: StataCorp LLC. Abgerufen von http://statmodel.com/download/Bayes3.pdf.

[r3] Asparouhov, T., & Muthén, B. (2020). Comparison of models for the analysis of intensive longitudinal data. Structural Equation Modeling: A Multidisciplinary Journal, 27(2), 275–297. 10.1080/10705511.2019.1626733

[r4] Bell, I. H., Eisner, E., Allan, S., Cartner, S., Torous, J., Bucci, S., & Thomas, N. (2023). Methodological characteristics and feasibility of ecological momentary assessment studies in psychosis: a systematic review and meta-analysis. Schizophrenia Bulletin, sbad127. 10.1093/schbul/sbad127 PMC10919779 37606276

[r5] Bogudzińska, B., Jaworski, A., Zajdel, A., Skrzypek, K., & Misiak, B. (2024). The experience sampling methodology in psychosis risk states: A systematic review. Journal of Psychiatric Research, 175, 34–41. 10.1016/j.jpsychires.2024.04.050 38704979

[r59] Borsboom, D., & Haslbeck, J. (2024). Integrating Intra- and Interindividual Phenomena in Psychological Theories. Multivariate Behavioral Research, 1–20. 10.1080/00273171.2024.2336178. 38989982

[r6] Cole, D. A., & Maxwell, S. E. (2003). Testing mediational models with longitudinal data: questions and tips in the use of structural equation modeling. Journal of Abnormal Psychology, 112(4), 558–577. 10.1037/0021-843X.112.4.558 14674869

[r7] Cornblatt, B. A., Carrión, R. E., Auther, A., McLaughlin, D., Olsen, R. H., John, M., & Correll, C. U. (2015). Psychosis prevention: a modified clinical high risk perspective from the recognition and prevention (RAP) Program. *American Journal of Psychiatry*, 172(10), 986–994. 10.1176/appi.ajp.2015.13121686 26046336 PMC4993209

[r8] Cunningham, T., Hoy, K., & Shannon, C. (2016). Does childhood bullying lead to the development of psychotic symptoms? A meta-analysis and review of prospective studies. Psychosis, 8(1), 48–59. 10.1080/17522439.2015.1053969

[r9] Domingos, P. (1999). The role of occam’s razor in knowledge discovery. Data Mining and Knowledge Discovery, 3(4), 409–425. 10.1023/A:1009868929893

[r10] Epskamp, S., Van Borkulo, C. D., Van Der Veen, D. C., Servaas, M. N., Isvoranu, A.-M., Riese, H., & Cramer, A. O. J. (2018). Personalized network modeling in psychopathology: the importance of contemporaneous and temporal connections. Clinical Psychological Science, 6(3), 416–427. 10.1177/2167702617744325 29805918 PMC5952299

[r11] Fett, A.-K. J., Lemmers-Jansen, I. L. J., & Krabbendam, L. (2019). Psychosis and urbanicity: A review of the recent literature from epidemiology to neurourbanism. Current Opinion in Psychiatry, 32(3), 232–241. 10.1097/YCO.0000000000000486 30724751 PMC6493678

[r12] Fusar-Poli, P., Borgwardt, S., Bechdolf, A., Addington, J., Riecher-Rössler, A., Schultze-Lutter, F., Yung, A. (2013). The psychosis high-risk state: a comprehensive state-of-the-art review. JAMA Psychiatry, 70(1), 107. 10.1001/jamapsychiatry.2013.269 23165428 PMC4356506

[r13] Fusar-Poli, P., Salazar De Pablo, G., Correll, C. U., Meyer-Lindenberg, A., Millan, M. J., Borgwardt, S., Arango, C. (2020). Prevention of psychosis: advances in detection, prognosis, and intervention. JAMA Psychiatry, 77(7), 755. 10.1001/jamapsychiatry.2019.4779 32159746

[r14] Fux, L., Walger, P., Schimmelmann, B. G., & Schultze-Lutter, F. (2013). The schizophrenia proneness instrument, child and youth version (SPI-CY): Practicability and discriminative validity. Schizophrenia Research, 146(1–3), 69–78. 10.1016/j.schres.2013.02.014 23473813

[r15] GBD 2019 Mental Disorders Collaborators (2022). Global, regional, and national burden of 12 mental disorders in 204 countries and territories, 1990–2019: A systematic analysis for the Global Burden of Disease Study 2019. The Lancet Psychiatry, 9(2), 137–150. 10.1016/S2215-0366(21)00395-3 35026139 PMC8776563

[r16] Granger, C. W. J. (1969). Investigating causal relations by econometric models and cross-spectral methods. Econometrica, 37(3), 424. 10.2307/1912791

[r17] Hallquist, M. N., & Wiley, J. F. (2018). *MplusAutomation*: An R package for facilitating large-scale latent variable analyses in M *plus*. Structural Equation Modeling: A Multidisciplinary Journal, 25(4), 621–638. 10.1080/10705511.2017.1402334 30083048 PMC6075832

[r18] Hamaker, E. L., Kuiper, R. M., & Grasman, R. P. P. P. (2015). A critique of the cross-lagged panel model. Psychological Methods, 20(1), 102–116. 10.1037/a0038889 25822208

[r19] Judge, A. M., Estroff, S. E., Perkins, D. O., & Penn, D. L. (2008). Recognizing and responding to early psychosis: a qualitative analysis of individual narratives. Psychiatric Services, 59(1), 96–99. 10.1176/ps.2008.59.1.96 18182546

[r20] Klippel, A., Myin-Germeys, I., Chavez-Baldini, U., Preacher, K. J., Kempton, M., Valmaggia, L., Reininghaus, U. (2017). Modeling the interplay between psychological processes and adverse, stressful contexts and experiences in pathways to psychosis: an experience sampling study. Schizophrenia Bulletin, 43(2), 302–315. 10.1093/schbul/sbw185 28204708 PMC5605264

[r21] Klippel, A., Schick, A., Myin-Germeys, I., Rauschenberg, C., Vaessen, T., & Reininghaus, U. (2022). Modelling the temporal interplay between stress and affective disturbances in pathways to psychosis: An experience sampling study. Psychological Medicine, 52(13), 2776–2785. 10.1017/S0033291720004894 33678198 PMC9647515

[r22] Klippel, A., Viechtbauer, W., Reininghaus, U., Wigman, J., Van Borkulo, C., MERGE, … Wichers, M. (2018). The cascade of stress: A network approach to explore differential dynamics in populations varying in risk for psychosis. Schizophrenia Bulletin, 44(2), 328–337. 10.1093/schbul/sbx037 28338969 PMC5815145

[r23] Kramer, I., Simons, C. J. P., Wigman, J. T. W., Collip, D., Jacobs, N., Derom, C., … Wichers, M. (2014). Time-lagged moment-to-moment interplay between negative affect and paranoia: New insights in the affective pathway to psychosis. Schizophrenia Bulletin, 40(2), 278–286. 10.1093/schbul/sbs194 23407984 PMC3932075

[r24] Lucas, R. E. (2023). Why the cross-lagged panel model is almost never the right choice. Advances in Methods and Practices in Psychological Science, 6(1), 25152459231158378. 10.1177/25152459231158378

[r25] Ma, C. F., Chan, S. K. W., Chung, Y. L., Ng, S. M., Hui, C. L. M., Suen, Y. N., & Chen, E. Y. H. (2021). The predictive power of expressed emotion and its components in relapse of schizophrenia: A meta-analysis and meta-regression. Psychological Medicine, 51(3), 365–375. 10.1017/S0033291721000209 33568244

[r26] McGlashan, T., Walsh, B., & Woods, S. (2010). The psychosis-risk syndrome: Handbook for diagnosis and follow-up. Oxford University Press.

[r27] McGorry, P. D., Mei, C., Dalal, N., Alvarez-Jimenez, M., Blakemore, S.-J., Browne, V., Killackey, E. (2024). The lancet psychiatry commission on youth mental health. The Lancet Psychiatry, 11(9), 731–774. 10.1016/S2215-0366(24)00163-9 39147461

[r28] McNeish, D., & Hamaker, E. L. (2020). A primer on two-level dynamic structural equation models for intensive longitudinal data in Mplus. Psychological Methods, 25(5), 610–635. 10.1037/met0000250 31855015

[r29] Meyer, R. (2016). Deviance Information Criterion (DIC ). In R. S. Kenett, N. T. Longford, W. W. Piegorsch, & F. Ruggeri (Hrsg.), Wiley StatsRef: Statistics Reference Online (1. Aufl., S. 1–6). Wiley. 10.1002/9781118445112.stat07878

[r30] Michel, C., Kaess, M., Flückiger, R., Büetiger, J. R., Schultze‐Lutter, F., Schimmelmann, B. G., Kindler, J. (2022). The Bern early recognition and Intervention Centre for mental crisis (FETZ Bern)—An 8‐year evaluation. Early Intervention in Psychiatry, 16(3), 289–301. 10.1111/eip.13160 33960114

[r31] Michel, C., Lerch, S., Büetiger, J. R., Flückiger, R., Cavelti, M., Koenig, J., … Kindler, J. (2022). An ecological momentary assessment study of age effects on perceptive and non-perceptive clinical high-risk symptoms of psychosis. European Child & Adolescent Psychiatry. 10.1007/s00787-022-02003-9 PMC9116495 35585271

[r32] Michel, C., Schimmelmann, B. G., Kupferschmid, S., Siegwart, M., & Schultze-Lutter, F. (2014). Reliability of telephone assessments of at-risk criteria of psychosis: A comparison to face-to-face interviews. Schizophrenia Research, 153(1–3), 251–253. 10.1016/j.schres.2014.01.025 24529611

[r33] Molenberghs, G., & Verbeke, G. (2007). Likelihood ratio, score, and Wald tests in a constrained parameter space. The American Statistician, 61(1), 22–27. 10.1198/000313007X171322

[r34] Monsonet, M., Rockwood, N. J., Kwapil, T. R., & Barrantes-Vidal, N. (2022). Psychological pathways to Paranoia and psychotic-like experiences in daily-life: The mediating role of distinct affective disturbances. Schizophrenia Bulletin, 48(5), 1053–1065. 10.1093/schbul/sbac071 35759215 PMC9434429

[r35] Muthén, B., & Asparouhov, T. (2024). Can cross-lagged panel modeling be relied on to establish cross-lagged effects? The case of contemporaneous and reciprocal effects. Psychological Methods. 10.1037/met0000661 38815066

[r36] Muthén, L. K., & Muthén, B. O. (2017). Mplus User’s Guide. Eighth Edition. Los Angeles, CA: Muthén & Muthén.

[r37] Myin-Germeys, I., Kasanova, Z., Vaessen, T., Vachon, H., Kirtley, O., Viechtbauer, W., & Reininghaus, U. (2018). Experience sampling methodology in mental health research: New insights and technical developments. World Psychiatry, 17(2), 123–132. 10.1002/wps.20513 29856567 PMC5980621

[r38] Myin-Germeys, I., & van Os, J. (2007). Stress-reactivity in psychosis: Evidence for an affective pathway to psychosis. Clinical Psychology Review, 27(4), 409–424. 10.1016/j.cpr.2006.09.005 17222489

[r39] Nuechterlein, K. H., Dawson, M. E., Ventura, J., Gitlin, M., Subotnik, K. L., Snyder, K. S., Bartzokis, G. (1994). The vulnerability/stress model of schizophrenic relapse: A longitudinal study. Acta Psychiatrica Scandinavica, 89(s382), 58–64. 10.1111/j.1600-0447.1994.tb05867.x 8091999

[r40] Paetzold, I., Myin-Germeys, I., Schick, A., Nelson, B., Velthorst, E., Schirmbeck, F., Reininghaus, U. (2021). Stress reactivity as a putative mechanism linking childhood trauma with clinical outcomes in individuals at ultra-high-risk for psychosis: Findings from the EU-GEI High Risk Study. Epidemiology and Psychiatric Sciences, 30, e40. 10.1017/S2045796021000251 34044905 PMC8193966

[r41] R Core Team. (2023). R: A Language and Environment for Statistical Computing. Vienna, Austria: R Foundation for Statistical Computing. Abgerufen von https://www.R-project.org/.

[r42] Radley, J., Barlow, J., & Johns, L. C. (2022). Parenting and psychosis: An experience sampling methodology study investigating the inter‐relationship between stress from parenting and positive psychotic symptoms. British Journal of Clinical Psychology, 61(4), 1236–1258. 10.1111/bjc.12389 35938517 PMC9804428

[r43] Rauschenberg, C., Van Os, J., Goedhart, M., Schieveld, J. N. M., & Reininghaus, U. (2021). Bullying victimization and stress sensitivity in help-seeking youth: Findings from an experience sampling study. European Child & Adolescent Psychiatry, 30(4), 591–605. 10.1007/s00787-020-01540-5 32405792 PMC8041697

[r44] Reininghaus, U., Depp, C. A., & Myin-Germeys, I. (2016). Ecological Interventionist Causal Models in Psychosis: Targeting Psychological Mechanisms in Daily Life. Schizophrenia Bulletin, 42(2), 264–269. 10.1093/schbul/sbv193 26707864 PMC4753613

[r45] Reininghaus, U., Gayer-Anderson, C., Valmaggia, L., Kempton, M. J., Calem, M., Onyejiaka, A., Morgan, C. (2016). Psychological processes underlying the association between childhood trauma and psychosis in daily life: An experience sampling study. Psychological Medicine, 46(13), 2799–2813. 10.1017/S003329171600146X 27400863 PMC5358473

[r46] Reininghaus, U., Kempton, M. J., Valmaggia, L., Craig, T. K. J., Garety, P., Onyejiaka, A., Morgan, C. (2016). Stress sensitivity, aberrant salience, and threat anticipation in early psychosis: An experience sampling study. Schizophrenia Bulletin, 42(3), 712–722. 10.1093/schbul/sbv190 26834027 PMC4838104

[r47] Schick, A., Van Winkel, R., Lin, B. D., Luykx, J. J., De Zwarte, S. M. C., Van Eijk, K. R., Reininghaus, U. (2023). Polygenic risk, familial liability and stress reactivity in psychosis: An experience sampling study. Psychological Medicine, 53(7), 2798–2807. 10.1017/S0033291721004761 34991751 PMC10235643

[r48] Schimmelmann, B. G., Michel, C., Martz-Irngartinger, A., Linder, C., & Schultze-Lutter, F. (2015). Age matters in the prevalence and clinical significance of ultra-high-risk for psychosis symptoms and criteria in the general population: Findings from the BEAR and BEARS-kid studies. World Psychiatry, 14(2), 189–197. 10.1002/wps.20216 26043337 PMC4471976

[r49] Schultze-Lutter, F., Addington, J., Ruhrmann, S., & Klosterkötter, J. (2007). Schizophrenia proneness instrument, adult version (SPI-A). Rome*:* Giovanni Fioriti.

[r50] Schultze-Lutter, F., Michel, C., Schmidt, S. J., Schimmelmann, B. G., Maric, N. P., Salokangas, R. K. R., Klosterkötter, J. (2015). EPA guidance on the early detection of clinical high risk states of psychoses. European Psychiatry, 30(3), 405–416. 10.1016/j.eurpsy.2015.01.010 25735810

[r51] Schultze-Lutter, F., Ruhrmann, S., Michel, C., Kindler, J., Schimmelmann, B. G., & Schmidt, S. J. (2020). Age effects on basic symptoms in the community: A route to gain new insight into the neurodevelopment of psychosis? European Archives of Psychiatry and Clinical Neuroscience, 270(3), 311–324. 10.1007/s00406-018-0949-4 30361925 PMC7069926

[r52] Shah, J. L., Jones, N., van Os, J., McGorry, P. D., & Gülöksüz, S. (2022). Early intervention service systems for youth mental health: Integrating pluripotentiality, clinical staging, and transdiagnostic lessons from early psychosis. The Lancet Psychiatry, 9(5), 413–422. 10.1016/S2215-0366(21)00467-3 35430004

[r53] Shapiro, D. I., Li, H., Kline, E. R., & Niznikiewicz, M. A. (2019). Assessment of Risk for Psychosis. In H. Li, D. I. Shapiro, & L. J. Seidman (Hrsg.), Handbook of attenuated psychosis syndrome across cultures (7–40). Cham: Springer International Publishing. 10.1007/978-3-030-17336-4_2

[r54] Trotta, A., Murray, R. M., & Fisher, H. L. (2015). The impact of childhood adversity on the persistence of psychotic symptoms: A systematic review and meta-analysis. Psychological Medicine, 45(12), 2481–2498. 10.1017/S0033291715000574 25903153

[r55] Uhlhaas, P. J., Davey, C. G., Mehta, U. M., Shah, J., Torous, J., Allen, N. B., Wood, S. J. (2023). Towards a youth mental health paradigm: A perspective and roadmap. Molecular Psychiatry, 28(8), 3171–3181. 10.1038/s41380-023-02202-z 37580524 PMC10618105

[r56] Van Der Steen, Y., Gimpel‐Drees, J., Lataster, T., Viechtbauer, W., Simons, C. J. P., Lardinois, M., Myin‐Germeys, I. (2017). Clinical high risk for psychosis: The association between momentary stress, affective and psychotic symptoms. Acta Psychiatrica Scandinavica, 136(1), 63–73. 10.1111/acps.12714 28260264

[r57] Van Os, J., & Linscott, R. J. (2012). Introduction: The extended psychosis phenotype--relationship with schizophrenia and with ultrahigh risk status for psychosis. Schizophrenia Bulletin, 38(2), 227–230. 10.1093/schbul/sbr188 22355185 PMC3283160

[r58] Woods, S. W., Walsh, B. C., Powers, A. R., & McGlashan, T. H. (2019). Reliability, validity, epidemiology, and cultural variation of the structured interview for psychosis-risk syndromes (sips) and the scale of psychosis-risk symptoms (SOPS). In H. Li, D. I. Shapiro, & L. J. Seidman (Hrsg.), Handbook of attenuated psychosis syndrome across cultures (85–113). Cham: Springer International Publishing. 10.1007/978-3-030-17336-4_5

