# Supplementary material for: The relationship between stress and clinical high-risk symptoms of psychosis in daily life: impact of contemporaneous paths on cross-lagged effects
Source: Psychol Med. 2025 Mar 3;55:e68. doi: 10.1017/S0033291725000364 (PMC12080662; doi:10.1017/S0033291725000364)
Supplement: Cavelti et al. supplementary material [file S0033291725000364sup001.pdf]

Supplementary Materials for:

The relationship between stress and clinical high-risk symptoms of psychosis in daily life: Impact of contemporaneous paths on cross-lagged effects

Submitted to:

Psychological Medicine

Marialuisa Cavelti[1], Janko M. Kaeser[1], Silvano Sele[1], Thomas Berger[2], Michael Kaess[1,3], Jochen Kindler[1], Chantal Michel[1]

[1] University Hospital of Child and Adolescent Psychiatry and Psychotherapy, University of Bern, Bern, Switzerland

[2] Department of Clinical Psychology and Psychotherapy, University of Bern, Bern, Switzerland

[3] Department of Child and Adolescent Psychiatry, Centre for Psychosocial Medicine, University of Heidelberg, Heidelberg, Germany

Corresponding author: Marialuisa Cavelti, University Hospital for Child and Adolescent Psychiatry and Psychotherapy, Bolligenstrasse 111, 3000 Bern 60, Switzerland, marialuisa.cavelti@unibe.ch

Content

The clinical high-risk state of psychosis (CHR-P): Two complementary approaches 3
Additional information on Ecological Momentary Assessment (EMA) 5
Detailed description of the methodological approach 5
Items translated from German to English 6
Evaluation of Items' psychometric properties 7
EMA adherence: Additional Information on the Distribution 8
Individual EMA Trajectories of CHR-P Symptoms 10
Descriptive statistics of the prompt-to-response duration 11
Correlation Matrix of Adherence, Age and Severity of Symptoms 13
Potential reasons for EMA missing data 13
Age: Additional information on the distribution 16
Full report of Model C with random effects 18
Full report of the moderation model 20
Commentary 22
Full report of the model with CHR symptoms split into APS/B(L)IPS and BS 24
Commentary 27
Full report of the model with CHR symptoms split into perceptive versus non-perceptive symptoms 28
Commentary 31
Mplus code for conducting R-DSEM 32
Evaluating model structures with simulated data from a lag0 model structure without cross-lagged effects (Monte Carlo Simulation) 44
Fitting true model 44
Fitting covariance between residuals (instead of lag0) 54
Fitting wrongly directed lag0 64
References 74

The clinical high-risk state of psychosis (CHR-P): Two complementary approaches

The CHR-P refers to a condition characterized by the presence of early symptoms or markers that suggest an increased likelihood of developing a psychotic disorder. CHR-P criteria aim to identify individuals who are at risk of transitioning to a first episode of psychosis, facilitating early intervention to potentially delay or prevent progression. Currently, two complementary approaches are used to define and characterize the CHR-P state: the ultra-high risk (UHR) and the basic symptoms (BS) criteria (Fusar-Poli et al., 2013; Klosterkotter, Schultze-Lutter, Bechdolf, & Ruhrmann, 2011; Schultze-Lutter et al., 2015). While BS criteria were developed to detect the risk for psychosis as early as possible in the development of the illness, ideally before functional impairments appeared, UHR criteria were originally developed with the explicit aim of detecting an imminent risk for psychoses, i.e., persons at risk for developing a first-episode within the next 12 months (Keshavan, DeLisi, & Seidman, 2011; Klosterkotter et al., 2011; Phillips, Yung, & McGorry, 2000). Given the complementary nature of the two concepts, there is a growing trend in clinical practice to apply both sets of CHR-P criteria – UHR and BS – simultaneously (Fusar-Poli, Borgwardt, & Valmaggia, 2008).

UHR criteria

To meet UHR criteria, the presence of one or more of the following conditions: attenuated psychotic symptoms (APS), brief (limited) intermittent psychotic symptoms (B(L)IPS), and/or trait vulnerability including a substantial decline in psychosocial functioning (genetic risk and functional decline [GRFD]; Schultze-Lutter et al., 2015). APS are characterized by delusions, hallucinations, or disorganized speech that are present in an attenuated form. Reality testing is intact, but symptoms are of sufficient severity and/or frequency to potentially cause distress that might result in help-seeking behaviour (Fusar-Poli, Raballo, & Parnas, 2017; Shrivastava et al., 2011). In contrast, individuals with B(L)IPS experience a self-remitting psychotic episode with short-lived full-blown psychotic symptoms (Fusar-Poli et al., 2016). UHR criteria were originally developed to detect individuals at imminent risk of psychosis, defined as a high likelihood of experiencing a first psychotic episode within the next 12 months (Lisa J. Phillips, Yung, & McGorry, 2000). Compared to BS criteria, UHR criteria are more commonly applied in clinical research (Fusar-Poli et al., 2013; Ruhrmann et al., 2010; Yung et al., 2006).

BS criteria

In contrast to UHR criteria, the BS criteria, which include the partially overlapping "cognitive-perceptive basic symptoms" (COPER; see symptom list in Table 1 and "cognitive disturbances" (COGDIS; see symptom list in Table 2), focus on identifying the earliest possible specific symptom, ideally before significant functional impairments arise (Schultze-Lutter, 2009; Frauke Schultze-Lutter et al., 2016). COPER and COGDIS encompass subtle self-experienced disturbances in speech, thinking as well as acoustic and visual perception processes. Compared to the premorbid self, these symptoms are experienced as different from "normal" mental states. These disturbances are clearly distinguishable from experiences ascribed to schizotypy or trait-like subtle disturbances reported in genetic high-risk individuals (Debbané et al., 2015; Jones, 2002; Parnas & Carter, 2002).

Table 1 of SM

At-risk Criterion Cognitive-Perceptive Basic Symptoms (COPER)

thought interference (C2)[a]
thought perseveration (O1)
thought pressure (D3)
thought blockages (C3)
disturbance of receptive speech (C4)
decreased ability to discriminate between ideas and perception, fantasy and true memories (O2)
unstable ideas of reference (D4)
derealisation (O8)
visual perception disturbances (D5, F2, F3, O4)
acoustic perception disturbances (F5, O5)

Notes. Adapted from Frauke Schultze-Lutter et al. (2012)

[a] Item numbers refer to the SPI-A (Frauke Schultze-Lutter et al., 2007)

Table 2 of SM

High-risk Criterion Cognitive Disturbances (COGDIS)

inability to divide attention (B1)[a]
thought interference (C2)
thought pressure (D3)
thought blockages (C3)
disturbance of receptive speech (C4)
disturbance of expressive speech (C5)
unstable ideas of reference (D4)
disturbances of abstract thinking (O3)
captivation of attention by details of the visual field (O7)

Notes. Adapted from Frauke Schultze-Lutter et al. (2012)

[a] Item numbers refer to the SPI-A (Frauke Schultze-Lutter et al., 2007)

Additional information on Ecological Momentary Assessment (EMA)

Detailed description of the methodological approach

Participants received a smartphone with the movisensXS experience sampling application (Movisens GmbH, Karlsruhe, Germany), which served as an electronic diary. After instructions in the use of the application, the EMA assessment was conducted for seven consecutive days. Participants were prompted to fill out a total of eight assessments per day, randomly distributed between 8am and 10pm with a minimum of 25 minutes between prompts. To enhance compliance, participants could postpone each prompt once for 5, 10 or 15 minutes. After completing the EMA phase, participants were debriefed and compensated with CHF 10 for their participation in the EMA. At each EMA prompt, participants were asked to rate one item on their subjective stress level ("How stressed are you feeling right now?") as well as the frequency of the occurrence of 14 BS and seven APS/B(L)IPS since the last beep. Item selection for CHR symptoms was based on SPI-A/SPI-CY (Fux, Walger, Schimmelmann, & Schultze-Lutter, 2013; Frauke Schultze-Lutter, Addington, Ruhrmann, & Klosterkötter, 2007) as well as Appendix B of the SIPS (McGlashan, Walsh, & Woods, 2010) and guided by previous studies using EMA in psychosis research (Reininghaus et al., 2016). The wording of the items was shortened in order to limit the time required to complete the EMA to a maximum of five minutes. The EMA items for CHR symptoms were scored on a visual analog scale (VAS) ranging from 0 ('not at all') to 100 ('all the time'), with a higher score corresponding to a higher frequency and thus a higher severity of the symptom (see list of EMA items in the next section). The stress item was scored on a scale ranging from 0 ('minimally stressed') to 100 ('maximally stressed'). For analysis, we calculated for each EMA prompt 1) the mean score over all 21 CHR symptoms (mCHR); 2) separate mean scores for BS (BS items 1-14) and APS/B(L)IPS (APS/B(L)IPS items 1-7); and 3) separate mean scores for perceptive (PERC; BS Items 13, 14; APS/B(L)IPS items 1, 2, 5) and non-perceptive (NONP; BS items 1-12, APS/B(L)IPS items 3, 4, 6, 7) CHR symptoms.

Items translated from German to English

Basic symptoms (visual analog scale: 1 = not at all, 100 = all the time)

Since the last beep…

1) … I have found it difficult to divide my attention.
2) … my attention was captured by an unimportant detail in the environment.
3) … my concentration was disturbed by unimportant, irrelevant thoughts.
4) … my thoughts were interrupted or blocked.
5) … I had a chaos of unconnected thoughts in my head and could not suppress new thoughts.
6) … irrelevant thoughts or memories kept popping up and repeating themselves.
7) … I had problems understanding simple words or sentences.
8) … I had the feeling that I had to think longer to find the right words or sentences.
9) … I took everything to literally.
10) … I incorrectly related things from my surrounding to myself.
11) … I was unsure whether I had actually experienced something or just imagined it.
12) … the world seemed completely different, somehow fake, unreal and I felt like I was under a glass bell.
13) … my eyes played tricks on my and I saw familiar things in a different way.
14) … my eyes played tricks on my and I heard familiar things in a different way.

(Attenuated) psychotic symptoms (APS/B[L]IPS; visual analog scale: 1 = not at all, 100 = all the time)

Since the last beep…

1) … I have seen or perceived things that others did not see.
2) … I have heard sounds/voices that others did not hear.
3) … I have felt suspicious or skeptical of others.
4) … I could not control my own ideas or thoughts.
5) … my thoughts were so intense that I could almost hear them.
6) … I had ideas that others found unusual or strange.
7) … I was worried that something was wrong with my mind.

Subjective stress (visual analog scale: 1 = minimally stressed, 100 = maximally stressed)

1) How stressed do you feel right now?

Evaluation of Items' psychometric properties

The EMA items assessing CHR-P were derived from well-established interview measures described above to ensure appropriate wording to capture the phenomena of interest. We have calculated internal consistency for the two BS and APS mean scores as well as for the perceptive (PERC) and non-perceptive symptom (NONP) mean scores. First, we calculated the mean for each of the EMA items across prompts for each person. We then used these means to calculate Cronbach's Alpha, which was consistently excellent for all scores (BS: $\alpha_{std} = 0.954$, APS: $\alpha_{std} = 0.918$, PERC: $\alpha_{std} = 0.928$, NONP: $\alpha_{std} = 0.966$; Bland & Altman, 1997). As noted by Nezlek (2017), this represents the consistency of mean responses across occasions and not the occasion-level consistency. As of now, there is no gold standard on how internal consistency (i.e., reliability) should be assessed for EMA measures (Myin-Germeys & Kuppens, 2021). To provide further insight, we calculated correlation matrices at both the within- and the between-person level, accounting for the autoregressive effect of each variable (i.e., the lagged effect of a variable on itself; see this document, page 14 f.). The results indicate that items are correlated at both the within- and the between-person level, supporting the aggregation of item scores (i.e., mean or sum scores) across items.

In terms of convergent validity of our EMA items, an earlier study conducted with a largely overlapping sample ($N = 66$) demonstrated that interview scores predicted EMA ratings, with each one-point increase in interview scores corresponding to a 0.31 SD increase in the mean EMA rating ($\chi^2_{(1)} = 43.43$, $p < 0.001$, z = 6.59, $p < 0.001$; Michel et al., 2022). This significant small to medium agreement between interview and EMA ratings in our opinion underscores the validity of the EMA items, particularly given that the two methods assess psychosis risk symptoms through distinct approaches: EMA captures real-time symptom fluctuations that interviews relying on retrospective self-reports cannot measure.

EMA adherence: Additional Information on the Distribution

- Total of potentially answered prompts across all subjects: 7 days x 8 prompts x 79 subjects = 4’424
- Total answered EMA prompts across all subjects (N = 79): 3’063
- Average number of EMA prompts answered per subject: 38.77

Table 3 of SM

Descriptive Statistics of the EMA Adherence in %

Quantiles	.05	.10	.25	Mdn	.75	.90	.95
	25.00	32.14	50.00	75.00	91.07	96.43	98.21

Dispersion / Shape	Mean	Range	SD	SK	KT
	69.24	89.29	24.65	-0.62	-0.75

Extreme values	
lowest[a]	10.71 (2), 16.07, 25.00 (2), 30.36, 32.14 (3)
highest[a]	92.86 (2), 94.64 (3), 96.43 (5), 98.21 (4), 100.00 (2)

Notes. N = 79. SD = Standard Deviation. Mdn = Median. SK = Skewness. KT = Kurtosis.
[a] For frequencies greater than one, values are provided in parentheses.

Figure 1 of SM

Distribution of EMA adherence in %

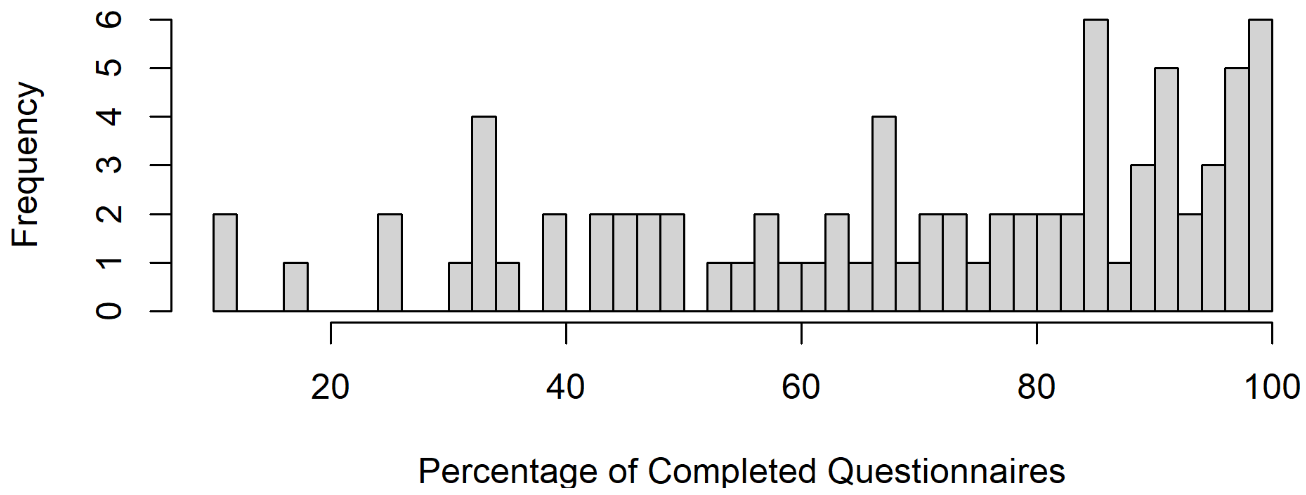

Notes. $N = 79$.

Individual EMA Trajectories of CHR-P Symptoms

Figure 2 of SM

Individual EMA Trajectories of CHR-P Symptoms

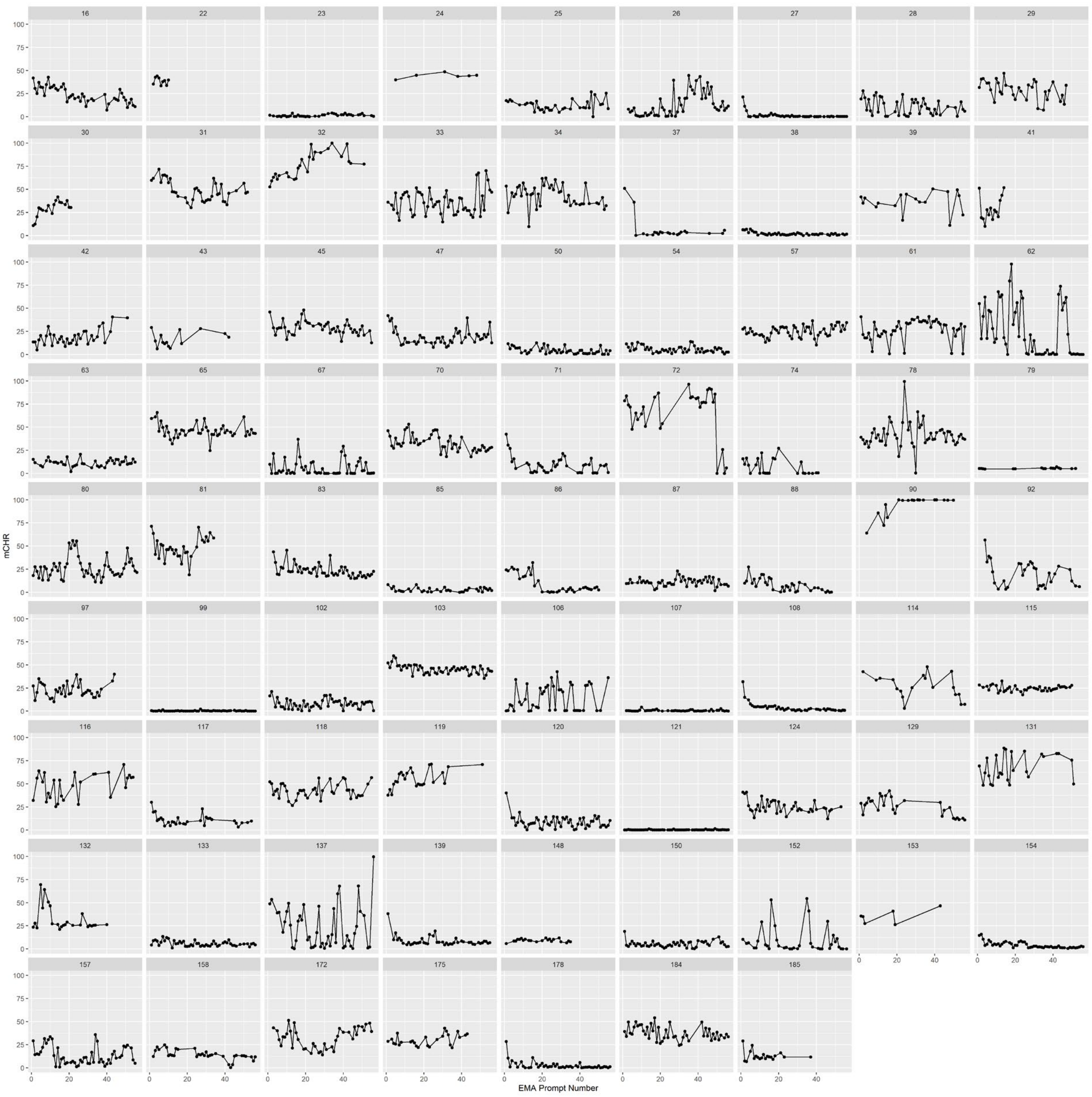

Note: N = 79. Number of observations = 3'063. mCHR represents the mean of CHR-P symptoms (Y-axis). "EMA Prompt Number" refers to prompts 1 to 56 sent to participants (X-axis).

Descriptive statistics of the prompt-to-response duration

Table 4 of SM

Descriptive Statistics of the Amount of Time from Prompt Signal to Answering of Prompt in Minutes

Quantiles	.05	.10	.25	Mdn	.75	.90	.95
	14.09	14.20	16.10	17.40	20.60	26.62	28.52
Dispersion / Shape	Mean	Range	SD	SK	KT		
	3.22	28.62	5.40	1.82	2.38		
Extreme values							
lowest[a]	0.03 (5), 0.05 (10), 0.07 (25), 0.08 (59), 0.10 (90)						
highest[a]	27.47, 27.52, 28.00, 28.32, 28.65						

Notes. N = 79. SD = Standard Deviation. Mdn = Median. SK = Skewness. KT = Kurtosis.
[a] For frequencies greater than one, values are provided in parentheses.

Figure 3 of SM

Distribution of the amount of time from prompt signal to answering of prompt in minutes

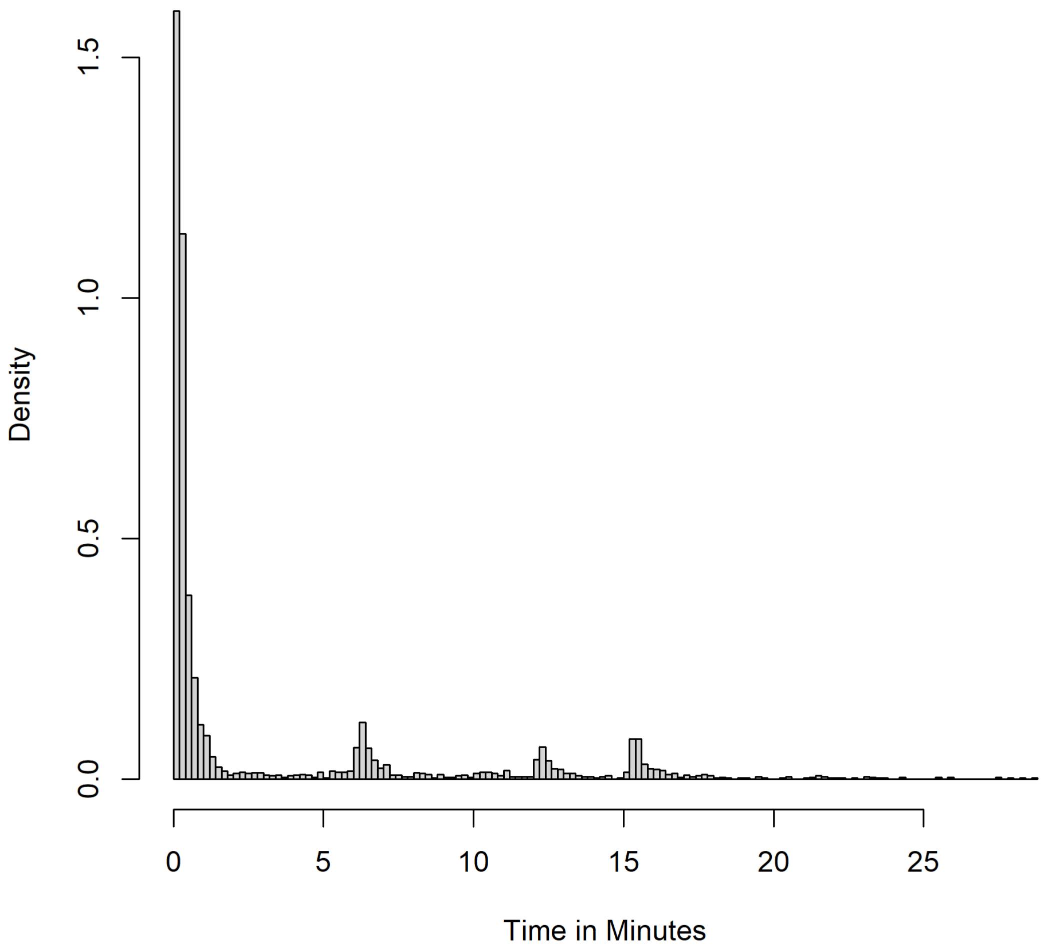

Notes. $N = 79$. Number of observations = 3'063.

Correlation Matrix of Adherence, Age and Severity of Symptoms

Figure 4 of SM

Correlation Matrix of Adherence, Age and Severity of Symptoms

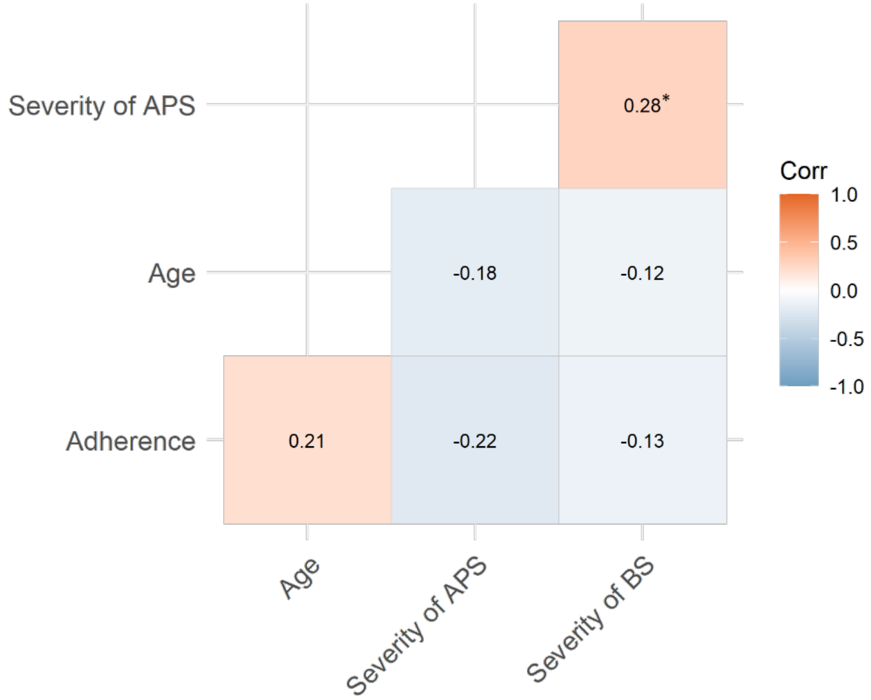

Notes. $N = 79$. '*' indicates $p < 0.05$. (A)PS = (Attenuated) Psychotic Symptoms. BS = Basic Symptoms. Adherence = Percentage of completed EMA questionnaires. Severity scores of APS and BS are based on clinical interviews.

Potential reasons for EMA missing data

We are not aware of any technical issues affecting the delivery of single prompts in our study. Participants were provided with a study smartphone with the movisensXS experience sampling application preinstalled. This application operates offline, ensuring no data loss due to connection problems. However, there were isolated cases of technical and practical challenges. For instance, one participant's phone was not working due to a software update, requiring a restart of the EMA phase with a replacement phone. Few participants reported forgetting the phone as a reason for low adherence. One participant returned the phone after two days, explaining that completing the questionnaires and focusing on symptoms worsened their condition. However, we decided to include the data of these two days with high adherence, as we deemed it more valuable to retain the available information than to exclude it. Overall, we were not able to identify any systematic reason for missing data (see also Figure 4 above for a correlation matrix of adherence, age and severity of symptoms).

Figure 5 of SM

Between-person Level Correlation Matrix

	ema_bs1	ema_bs2	ema_bs3	ema_bs4	ema_bs5	ema_bs6	ema_bs7	ema_bs8	ema_bs9	ema_bs10	ema_bs11	ema_bs12	ema_bs13	ema_bs14	ema_aps1	ema_aps2	ema_aps3	ema_aps4	ema_aps5	ema_aps6	ema_aps7
ema_bs1	1.00	0.85	0.90	0.90	0.83	0.80	0.73	0.73	0.59	0.58	0.46	0.35	0.44	0.54	0.42	0.39	0.59	0.66	0.64	0.72	0.69
ema_bs2	0.85	1.00	0.88	0.84	0.80	0.79	0.72	0.77	0.53	0.62	0.47	0.30	0.49	0.54	0.42	0.42	0.64	0.53	0.66	0.74	0.61
ema_bs3	0.90	0.88	1.00	0.91	0.90	0.91	0.65	0.73	0.57	0.61	0.45	0.32	0.43	0.49	0.42	0.41	0.67	0.70	0.74	0.72	0.72
ema_bs4	0.90	0.84	0.91	1.00	0.91	0.89	0.68	0.71	0.59	0.61	0.53	0.34	0.49	0.57	0.52	0.51	0.67	0.71	0.76	0.69	0.75
ema_bs5	0.83	0.80	0.90	0.91	1.00	0.93	0.62	0.63	0.52	0.63	0.60	0.31	0.42	0.57	0.52	0.49	0.65	0.72	0.73	0.68	0.74
ema_bs6	0.80	0.79	0.91	0.89	0.93	1.00	0.54	0.60	0.53	0.57	0.55	0.27	0.44	0.57	0.56	0.56	0.64	0.72	0.77	0.66	0.69
ema_bs7	0.73	0.72	0.65	0.68	0.62	0.54	1.00	0.90	0.75	0.72	0.52	0.42	0.57	0.65	0.42	0.40	0.66	0.54	0.52	0.65	0.63
ema_bs8	0.73	0.77	0.73	0.71	0.63	0.60	0.90	1.00	0.65	0.64	0.40	0.38	0.50	0.54	0.30	0.34	0.71	0.52	0.51	0.71	0.66
ema_bs9	0.59	0.53	0.57	0.59	0.52	0.53	0.75	0.65	1.00	0.75	0.58	0.42	0.67	0.79	0.58	0.55	0.74	0.62	0.69	0.66	0.64
ema_bs10	0.58	0.62	0.61	0.61	0.63	0.57	0.72	0.64	0.75	1.00	0.68	0.38	0.53	0.68	0.51	0.56	0.87	0.59	0.65	0.72	0.62
ema_bs11	0.46	0.47	0.45	0.53	0.60	0.55	0.52	0.40	0.58	0.68	1.00	0.50	0.59	0.73	0.66	0.65	0.64	0.65	0.54	0.55	0.64
ema_bs12	0.35	0.30	0.32	0.34	0.31	0.27	0.42	0.38	0.42	0.38	0.50	1.00	0.31	0.33	0.24	0.18	0.43	0.51	0.32	0.34	0.49
ema_bs13	0.44	0.49	0.43	0.49	0.42	0.44	0.57	0.50	0.67	0.53	0.59	0.31	1.00	0.85	0.79	0.63	0.57	0.58	0.48	0.55	0.57
ema_bs14	0.54	0.54	0.49	0.57	0.57	0.57	0.65	0.54	0.79	0.68	0.73	0.33	0.85	1.00	0.78	0.74	0.67	0.64	0.56	0.66	0.65
ema_aps1	0.42	0.42	0.42	0.52	0.52	0.56	0.42	0.30	0.58	0.51	0.66	0.24	0.79	0.78	1.00	0.87	0.54	0.64	0.53	0.48	0.61
ema_aps2	0.39	0.42	0.41	0.51	0.49	0.56	0.40	0.34	0.55	0.56	0.65	0.18	0.63	0.74	0.87	1.00	0.56	0.59	0.57	0.47	0.55
ema_aps3	0.59	0.64	0.67	0.67	0.65	0.64	0.66	0.71	0.74	0.87	0.64	0.43	0.57	0.67	0.54	0.56	1.00	0.66	0.68	0.80	0.76
ema_aps4	0.66	0.53	0.70	0.71	0.72	0.72	0.54	0.52	0.62	0.59	0.65	0.51	0.58	0.64	0.64	0.59	0.66	1.00	0.65	0.64	0.74
ema_aps5	0.64	0.66	0.74	0.76	0.73	0.77	0.52	0.51	0.69	0.65	0.54	0.32	0.48	0.56	0.53	0.57	0.68	0.65	1.00	0.67	0.56
ema_aps6	0.72	0.74	0.72	0.69	0.68	0.66	0.65	0.71	0.66	0.72	0.55	0.34	0.55	0.66	0.48	0.47	0.80	0.64	0.67	1.00	0.68
ema_aps7	0.69	0.61	0.72	0.75	0.74	0.69	0.63	0.66	0.64	0.62	0.64	0.49	0.57	0.65	0.61	0.55	0.76	0.74	0.56	0.68	1.00

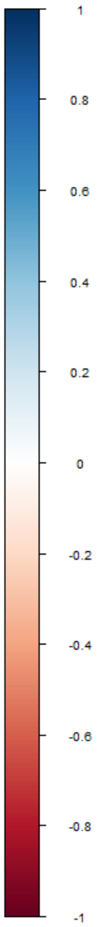

Note. N = 79. Number of observations = 3'063. Labels correspond to item list on page 6.

Figure 6 of SM

Within-person Level Correlation Matrix

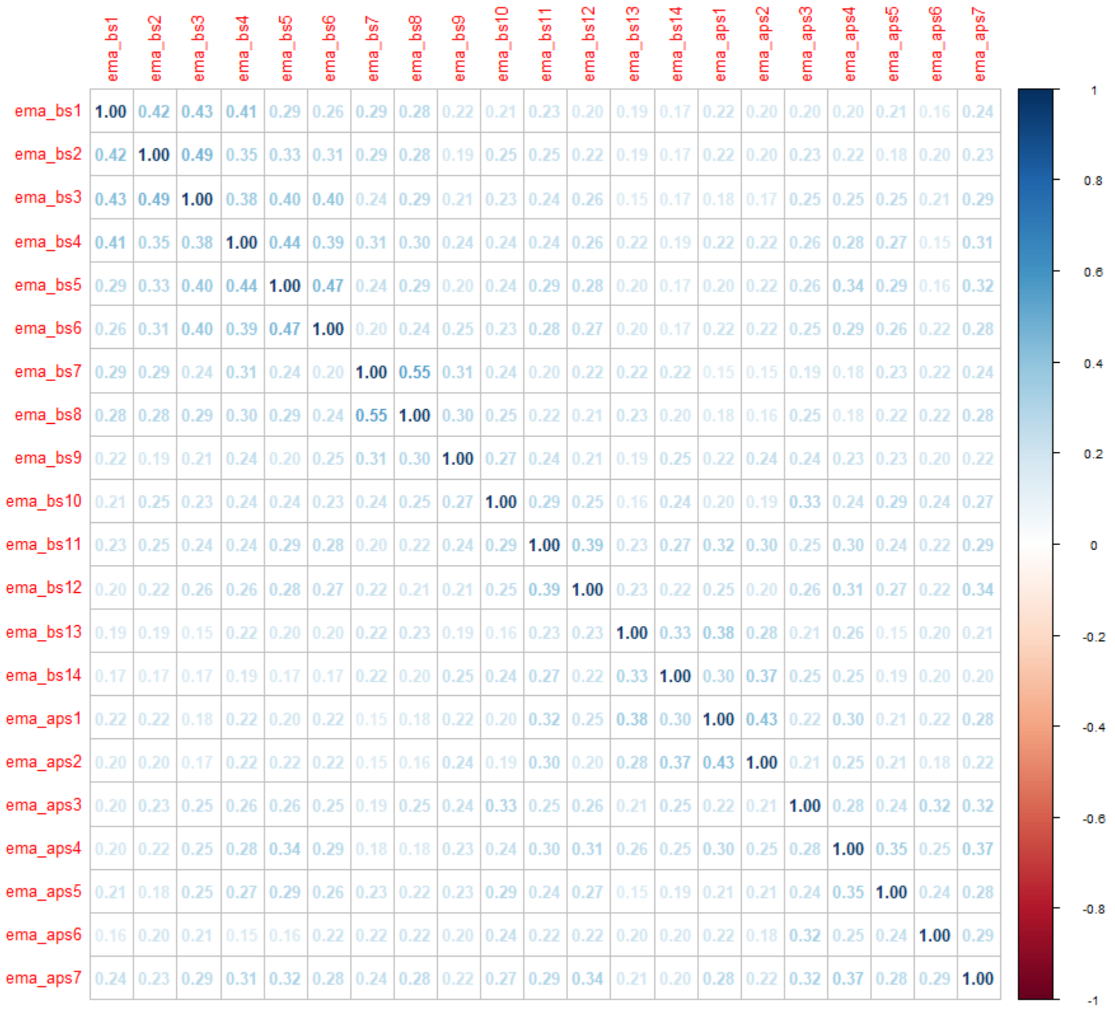

Note. N = 79. Number of observations = 3'063. Labels correspond to item list on page 6.

Age: Additional information on the distribution

Table 5 of SM

Descriptive Statistics of the Age of the Final Sample

Quantiles	.05	.10	.25	Mdn	.75	.90	.95
	14.09	14.20	16.10	17.40	20.60	26.62	28.52
Dispersion / Shape	Mean	Range	SD	SK	KT		
	18.99	24.90	4.93	1.55	2.45		
Extreme values							
lowest[a]	11.5, 13.1, 13.4, 14.0, 14.1 (2)						
highest[a]	28.2, 28.5, 28.7, 30.9, 36.4 (2)						

Notes. N = 79. SD = Standard Deviation. Mdn = Median. SK = Skewness. KT = Kurtosis.
[a] For frequencies greater than one, values are provided in parentheses.

Figure 7 of SM

Distribution of Age

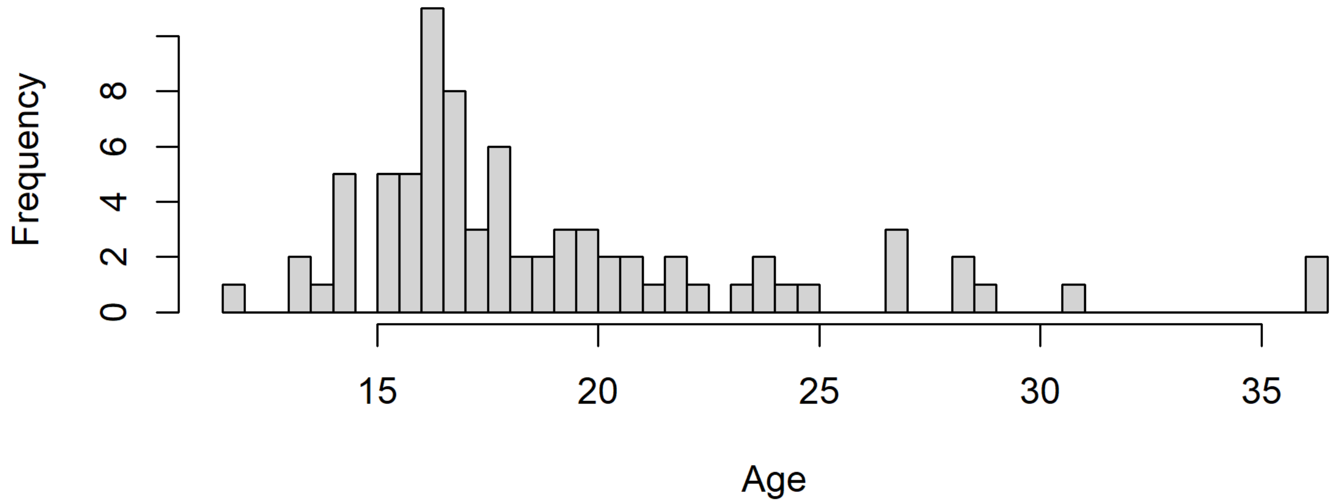

Notes. $N = 79$.

Full report of Model C with random effects

Table 6 of SM
Results of Model C with random effects

**Model A**						
Within-Level standardized estimates averaged across clusters		Estimate	SD	P^c	LCI	UCI
**Effects**						
mCHR (T-1) → Stress (T)		0.017	0.022	0.218	-0.026	0.058
Stress (T-1) → mCHR (T)		0.015	0.009	0.043	-0.003	0.032
**Effects with random slopes (rs)**						
rsStress:	Stress (T-1) → Stress (T)	0.387*	0.031	0.000	0.325	0.445
rsCHR:	mCHR (T-1) → mCHR (T)	0.514*	0.022	0.000	0.467	0.552
rsLag0:	mCHR (T) → Stress (T)	0.271*	0.023	0.000	0.226	0.315
**Random residual variance[d]**						
logvStress:	logv for Stress	0.676*	0.019	0.000	0.638	0.712
logvCHR:	logv for mCHR	0.631*	0.017	0.000	0.598	0.663
**R^2**						
Stress		0.324*	0.019	0.000	0.288	0.362
mCHR		0.369*	0.017	0.000	0.337	0.402
Between Level		Estimate	SD	P^c	LCI	UCI
**Covariations**						
Stress ←→						
	rsStress	0.132	0.019	0.000	0.638	0.712
	rsCHR	-0.070	0.017	0.000	0.598	0.663
	rsLag0	0.177	0.175	0.236	-0.225	0.455
	logvStress	0.345*	0.144	0.326	-0.345	0.218
	logvCHR	0.141	0.171	0.171	-0.165	0.492
mCHR ←→						
	rsStress	-0.398*	0.159	0.010	-0.679	-0.066
	rsCHR	0.087	0.145	0.276	-0.200	0.354
	rsLag0	-0.114	0.173	0.249	-0.445	0.239
	logvStress	0.176	0.132	0.100	-0.095	0.420
	logvCHR	0.614*	0.081	0.000	0.437	0.754
rsStress ←→						
	rsCHR	0.082	0.081	0.000	0.437	0.754
	rsLag0	0.067	0.179	0.329	-0.262	0.431
	logvStress	0.129	0.222	0.388	-0.369	0.493
	logvCHR	-0.045	0.207	0.276	-0.302	0.495
rsCHR ←→						
	rsLag0	-0.244	0.136	0.111	-0.419	0.103

	logvStress	-0.166	0.143	0.128	-0.131	0.429
	logvCHR	0.171	0.143	0.084	-0.090	0.466
rsLag0 ←→						
	logvStress	0.207	0.220	0.168	-0.609	0.251
	logvCHR	-0.227	0.124	0.039	-0.030	0.453
logvStress ←→	logvCHR	0.237	0.118	0.003	0.106	0.573
Stress ←→	mCHR	0.365*	0.188	0.000	1.257	1.987
**Means**						
	Stress	1.616*	0.160	0.000	0.895	1.530
	mCHR	1.202*	0.345	0.000	0.972	2.315
	rsStress	1.511*	0.213	0.000	1.019	1.861
	rsCHR	1.424*	0.248	0.000	0.840	1.823
	rsLag0	1.289*	0.482	0.000	3.858	5.774
	logvStress	4.793*	0.195	0.000	1.379	2.138
	logvCHR	1.756*	0.000	0.000	1.000	1.000
**Residual Variances**						
	Stress	1.000	0.000	-	-	-
	mCHR	1.000	0.000	-	-	-
	rsStress	1.000	0.000	-	-	-
	rsCHR	1.000	0.000	-	-	-
	rsLag0	1.000	0.000	-	-	-
	logvStress	1.000	0.000	-	-	-
	logvCHR	1.000	0.000	-	-	-
Iterations[a]		2000				
PSR[b]		1.010				
DIC		195379.206				

Notes. *N* = 79. Number of observations = 3'063. All parameters in this table are standardized. mCHR = mean score over all CHR symptoms. LCI = lower part of confidence interval. UCI = upper part of confidence interval. DIC = Deviance Information Criterion. PSR = Potential Scale Reduction. 'A → B' symbolizes a directional effect of A on B (i.e., B regressed on A). 'A ←→ B' symbolizes an undirectional covariation between A and B.

[a] The minimum number of iterations was set at 2000. The model converged at this minimum.

[b] Value of the last iteration.

[c] P-value is one-tailed. Values below 0.025 are considered significant.

[d] The random residual variance (v) of normally distributed random effects has a lognormal distribution and is therefore called 'logv'.

252

Full report of the moderation model

Table 7 of SM

Results of Model C with random slopes and moderators on the between level

**Model A**						
Within-Level standardized estimates averaged over clusters		Estimate	SD	P[c]	LCI	UCI
**Effects**						
mCHR (T-1) → Stress (T)		0.014	0.021	0.273	-0.028	0.055
Stress (T-1) → mCHR (T)		0.016	0.009	0.055	-0.003	0.032
**Effects with random slopes (rs)**						
rsStress:	Stress (T-1) → Stress (T)	0.378*	0.030	0.000	0.320	0.435
rsCHR:	mCHR (T-1) → mCHR (T)	0.571*	0.023	0.000	0.522	0.613
rsLag0:	mCHR (T) → Stress (T)	0.268*	0.024	0.000	0.221	0.318
**Random residual variances[d]**						
logvStress:	logv for Stress	0.680*	0.019	0.000	0.643	0.716
logvCHR:	logv for mCHR	0.612*	0.019	0.000	0.574	0.649
**R^2**						
Stress		0.320*	0.019	0.000	0.284	0.357
mCHR		0.388*	0.019	0.000	0.351	0.426
Between Level		Estimate	SD	P[c]	LCI	UCI
**Effects of moderators on between level parameters**						
rsStress ←						
	Age	0.211*	0.098	0.015	0.012	0.394
	Sex	0.074	0.111	0.257	-0.152	0.285
	SevBS	0.008	0.115	0.468	-0.221	0.247
	SevAPS/B(L)IPS	-0.079	0.108	0.228	-0.289	0.134
rsCHR ←						
	Age	0.034	0.093	0.359	-0.156	0.214
	Sex	0.017	0.098	0.435	-0.179	0.208
	SevBS	-0.042	0.101	0.347	-0.237	0.162
	SevAPS/B(L)IPS	0.118	0.096	0.118	-0.071	0.302
rsCHR ←						
	Age	0.042	0.101	0.343	-0.160	0.239
	Sex	0.144	0.108	0.105	-0.069	0.345
	SevBS	-0.208	0.107	0.033	-0.405	0.012
	SevAPS/B(L)IPS	-0.078	0.109	0.237	-0.285	0.137
logvStress ←						
	Age	-0.063	0.086	0.249	-0.232	0.114
	Sex	0.011	0.087	0.450	-0.169	0.170

	SevBS	0.110	0.090	0.123	-0.071	0.281
	SevAPS/B(L)IPS	-0.040	0.086	0.329	-0.205	0.133
logvCHR ←						
	Age	0.015	0.079	0.428	-0.137	0.165
	Sex	-0.086	0.079	0.145	-0.233	0.073
	SevBS	0.265*	0.081	0.000	0.088	0.409
	SevAPS/B(L)IPS	0.207*	0.080	0.005	0.042	0.358
Stress ←						
	Age	0.007	0.085	0.468	-0.160	0.170
	Sex	-0.007	0.089	0.470	-0.180	0.168
	SevBS	0.132	0.087	0.071	-0.048	0.293
	SevAPS/B(L)IPS	-0.068	0.084	0.223	-0.227	0.105
mCHR ←						
	Age	-0.177*	0.077	0.013	-0.318	-0.025
	Sex	-0.147	0.079	0.041	-0.290	0.017
	SevBS	0.293*	0.081	0.001	0.122	0.435
	SevAPS/B(L)IPS	0.207*	0.074	0.003	0.061	0.350
**Covariations**						
Stress ←→						
	rsStress	0.140	0.181	0.223	-0.238	0.471
	rsCHR	-0.175	0.161	0.139	-0.489	0.142
	rsLag0	0.221	0.173	0.116	-0.156	0.521
	logvStress	0.303*	0.129	0.015	0.034	0.533
	logvCHR	0.108	0.131	0.213	-0.147	0.357
mCHR ←→						
	rsStress	-0.319	0.176	0.052	-0.616	0.065
	rsCHR	-0.016	0.164	0.468	-0.337	0.298
	rsLag0	0.065	0.171	0.359	-0.276	0.399
	logvStress	0.067	0.142	0.331	-0.212	0.345
	logvCHR	0.450*	0.114	0.000	0.187	0.643
rsStress ←→						
	rsCHR	0.277	0.188	0.079	-0.111	0.618
	rsLag0	0.024	0.230	0.463	-0.408	0.464
	logvStress	0.181	0.213	0.209	-0.254	0.546
	logvCHR	0.024	0.168	0.438	-0.296	0.345
rsCHR ←→						
	rsLag0	-0.203	0.193	0.164	-0.556	0.191
	logvStress	-0.197	0.142	0.090	-0.445	0.110
	logvCHR	0.002	0.158	0.495	-0.293	0.311

rsLag0 ←→						
	logvStress	0.279	0.140	0.032	-0.018	0.531
	logvCHR	-0.155	0.216	0.248	-0.526	0.310
logvStress ←→	logvCHR	0.209	0.128	0.055	-0.041	0.451
Stress ←→	mCHR	0.450*	0.112	0.000	0.210	0.638
**Intercepts**						
	Stress	1.410*	0.585	0.011	0.259	2.512
	mCHR	1.413*	0.528	0.007	0.333	2.397
	rsStress	0.368	0.696	0.296	-0.908	1.806
	rsCHR	1.614*	0.704	0.009	0.261	3.041
	rsLag0	1.364	0.749	0.033	-0.092	2.866
	logvStress	4.820*	0.725	0.000	3.404	6.245
	logvCHR	0.873	0.552	0.060	-0.199	1.960
**Residual Variances**						
	Stress	0.956*	0.034	0.000	0.869	0.993
	mCHR	0.801*	0.063	0.000	0.664	0.912
	rsStress	0.906*	0.056	0.000	0.768	0.981
	rsCHR	0.955*	0.037	0.000	0.856	0.993
	rsLag0	0.895*	0.064	0.000	0.735	0.980
	logvStress	0.959*	0.031	0.000	0.874	0.994
	logvCHR	0.861*	0.051	0.000	0.752	0.948
Iterations[a]		2000				
PSR[b]		1.005				
DIC		192949.109				

Notes. *N* = 78. Number of observations = 3’022. All parameters in this table are standardized. mCHR = mean score over all CHR symptoms. SevBS = Severity of BS, assessed by interview at baseline. SevAPS/B(L)IPS = Severity of APS/B(L)IPS, assessed by interview at baseline. LCI = lower part of confidence interval. UCI = upper part of confidence interval. DIC = Deviance Information Criterion. PSR = Potential Scale Reduction. 'A → B' symbolizes a directional effect of A on B (i.e., B regressed on A). 'A ←→ B' symbolizes an undirectional covariation between A and B.

[a] The minimum number of iterations was set at 2000. The model converged at this minimum.

[b] Value of the last iteration.

[c] P-value is one-tailed. Values below 0.025 are considered significant.

[d] The random residual variance (v) of normally distributed random effects has a lognormal distribution and is therefore called 'logv'.

Commentary

On the within level, age exerted a significant positive effect on the random autoregressive slope of stress (st.est: 0.211), meaning that the effect of $stress_{t-1}$ on $stress_t$ increased with age. There were no significant effects of the moderators on the autoregressive effect of mCHR and

the lag0-effect of $mCHR_t$ on $stress_t$. We additionally applied the Wald Test to jointly assess the impact of the moderators on the random slope of the lag0-effect. We failed to reject the null hypothesis ($p = 0.351$), indicating that the moderators did not significantly influence the contemporaneous path of $mCHR_t$ on $stress_t$. Further, APS/B(L)IPS and BS severities had both a significant positive effect on the random residual variance of mCHR ($APS/B(L)IPS_{st.est}$: 0.207; $BS_{st.est}$: 0.265), indicating that a higher symptom severity assessed via interview at baseline was associated with a greater intraindividual variance in CHR symptoms. On the between level, higher APS/B(L)IPS and BS severities were positively associated with a higher mCHR mean (i.e., across all measurement, $APS/B(L)IPS_{st.est}$: 0.207; $BS_{st.est}$: 0.293). In contrast, older age was associated with a lower mCHR (st.est: -0.177).

Full report of the model with CHR symptoms split into APS/B(L)IPS and BS

Of note: For the sake of simplicity, *APS/BL(I)PS* are referred to as *APS* in the table below.

Table 8 of SM

Results of Model C with CHR symptoms split into attenuated psychotic symptoms (APS) and basic symptoms (BS)

**Model A**						
Within-Level standardized estimates averaged across clusters		Estimate	SD	P^c	LCI	UCI
**Effects**						
APS (T-1) → Stress (T)		0.001	0.019	0.477	-0.036	0.039
BS (T-1) → Stress (T)		0.020	0.022	0.171	-0.026	0.064
BS (T-1) → APS (T)		0.273*	0.034	0.000	0.192	0.338
Stress (T-1) → APS (T)		0.014	0.013	0.150	-0.011	0.040
APS (T-1) → BS (T)		0.009	0.060	0.414	-0.080	0.153
Stress (T-1) → BS (T)		0.009	0.007	0.110	-0.005	0.022
**Effects with random slopes (rs)**						
rCov	APS ←→ BS	0.457*	0.017	0.000	0.421	0.488
rsStress:	Stress (T-1) → Stress (T)	0.389*	0.026	0.000	0.343	0.440
rsAPS:	APS (T-1) → APS (T)	0.217*	0.044	0.000	0.134	0.297
rsBS:	BS (T-1) → BS (T)	0.567*	0.040	0.000	0.479	0.624
rsAPSLag0:	APS (T) → Stress (T)	0.156*	0.031	0.000	0.087	0.213
rsBSLag0:	BS (T) → Stress (T)	0.136*	0.026	0.000	0.080	0.184
**Random residual variance[d]**						
logvStress:	logv for Stress	0.656*	0.019	0.000	0.616	0.691
logvAPS:	logv for APS	0.732*	0.021	0.000	0.691	0.773
logvBS:	logv for BS	0.620*	0.018	0.000	0.587	0.657
**R^2**						
Stress		0.344*	0.019	0.000	0.309	0.384
APS		0.268*	0.021	0.000	0.227	0.308
BS		0.380*	0.018	0.000	0.342	0.412
Between Level		Estimate	SD	P^c	LCI	UCI
**Covariations**						
Stress ←→						
	rsStress	0.138	0.186	0.236	-0.226	0.492
	rsAPS	-0.144	0.173	0.212	-0.462	0.215
	rsBS	-0.052	0.166	0.379	-0.372	0.281
	rsAPSLag0	-0.054	0.190	0.396	-0.394	0.346
	rsBSLag0	0.227	0.192	0.130	-0.194	0.557
	rCov	-0.083	0.151	0.308	-0.373	0.221

	logvStress	0.317*	0.131	0.013	0.042	0.547
	logvAPS	0.141	0.138	0.158	-0.143	0.397
	logvBS	0.088	0.139	0.278	-0.190	0.352
APS ←→						
	rsStress	-0.469*	0.174	0.011	-0.745	-0.073
	rsAPS	0.167	0.160	0.151	-0.162	0.467
	rsBS	0.108	0.155	0.249	-0.205	0.396
	rsAPSLag0	-0.257	0.158	0.066	-0.538	0.078
	rsBSLag0	0.038	0.192	0.423	-0.344	0.393
	rCov	0.059	0.145	0.347	-0.231	0.337
	logvStress	0.108	0.140	0.225	-0.175	0.373
	logvAPS	0.573*	0.093	0.000	0.368	0.726
	logvBS	0.457*	0.109	0.001	0.215	0.637
BS ←→						
	rsStress	-0.312	0.188	0.065	-0.631	0.098
	rsAPS	0.107	0.166	0.269	-0.228	0.419
	rsBS	0.108	0.162	0.266	-0.215	0.408
	rsAPSLag0	-0.228	0.165	0.093	-0.521	0.131
	rsBSLag0	0.049	0.195	0.406	-0.343	0.409
	rCov	0.053	0.149	0.362	-0.245	0.338
	logvStress	0.192	0.139	0.095	-0.091	0.444
	logvAPS	0.569*	0.095	0.000	0.359	0.727
	logvBS	0.545*	0.100	0.000	0.317	0.710
rsStress ←→						
	rsAPS	0.107	0.202	0.308	-0.284	0.488
	rsBS	0.245	0.202	0.131	-0.177	0.594
	rsAPSLag0	0.273	0.196	0.097	-0.157	0.614
	rsBSLag0	-0.149	0.225	0.264	-0.552	0.322
	rCov	0.117	0.176	0.276	-0.247	0.434
	logvStress	0.004	0.211	0.493	-0.403	0.425
	logvAPS	-0.149	0.165	0.191	-0.446	0.192
	logvBS	-0.057	0.169	0.375	-0.375	0.277
rsAPS ←→						
	rsBS	0.704*	0.119	0.000	0.407	0.873
	rsAPSLag0	0.106	0.193	0.299	-0.303	0.457
	rsBSLag0	-0.008	0.211	0.486	-0.406	0.418
	rCov	0.085	0.171	0.313	-0.258	0.412
	logvStress	-0.219	0.163	0.098	-0.520	0.123
	logvAPS	0.175	0.173	0.161	-0.174	0.497
	logvBS	0.092	0.176	0.296	-0.254	0.434
rsBS ←→						
	rsAPSLag0	0.008	0.205	0.485	-0.426	0.373

	rsBSLag0	-0.133	0.196	0.261	-0.485	0.273
	rCov	0.008	0.170	0.482	-0.315	0.338
	logvStress	-0.082	0.162	0.306	-0.390	0.243
	logvAPS	-0.023	0.158	0.442	-0.326	0.282
	logvBS	-0.064	0.155	0.346	-0.358	0.243
rsAPSLag0 ←→						
	rsBSLag0	-0.608	0.318	0.085	-0.913	0.199
	rCov	-0.020	0.211	0.464	-0.419	0.392
	logvStress	0.084	0.158	0.309	-0.237	0.379
	logvAPS	-0.301	0.239	0.126	-0.687	0.216
	logvBS	-0.261	0.233	0.150	-0.642	0.246
rsBSLag0 ←→						
	rCov	-0.145	0.210	0.257	-0.511	0.300
	logvStress	0.070	0.181	0.344	-0.267	0.452
	logvAPS	0.080	0.253	0.378	-0.411	0.567
	logvBS	-0.116	0.261	0.338	-0.590	0.419
rCov ←→						
	logvStress	-0.057	0.151	0.356	-0.350	0.239
	logvAPS	0.455*	0.124	0.001	0.180	0.664
	logvBS	0.476*	0.120	0.001	0.212	0.682
logvStress ←→						
	logvAPS	0.214	0.137	0.070	-0.068	0.464
	logvBS	0.179	0.137	0.106	-0.109	0.428
logvAPS ←→						
	logvBS	0.904*	0.029	0.000	0.835	0.946
Stress ←→						
	APS	0.311*	0.129	0.013	0.039	0.537
	BS	0.411*	0.120	0.001	0.149	0.614
APS ←→						
	BS	0.885*	0.034	0.000	0.801	0.934
**Means**						
	Stress	1.443*	0.183	0.000	1.088	1.813
	APS	0.913*	0.149	0.000	0.618	1.207
	BS	1.085*	0.159	0.000	0.775	1.408
	rsStress	1.426*	0.309	0.000	0.912	2.144
	rsAPS	0.769*	0.251	0.000	0.338	1.307
	rsBS	2.196*	0.351	0.000	1.580	2.955
	rsAPSLag0	0.446*	0.205	0.002	0.118	0.904
	rsBSLag0	0.458*	0.268	0.015	0.031	1.056
	rCov	1.121*	0.171	0.000	0.791	1.460

logvStress	4.302*	0.456	0.000	3.480	5.245
logvAPS	1.903*	0.220	0.000	1.485	2.356
logvBS	1.660*	0.199	0.000	1.268	2.053
**Residual Variances**					
Stress	1.000	0.000	-	-	-
APS	1.000	0.000	-	-	-
BS	1.000	0.000	-	-	-
rsStress	1.000	0.000	-	-	-
rsAPS	1.000	0.000	-	-	-
rsBS	1.000	0.000	-	-	-
rsAPSLag0	1.000	0.000	-	-	-
rsBSLag0	1.000	0.000	-	-	-
rCov	1.000	0.000			
logvStress	1.000	0.000	-	-	-
logvAPS	1.000	0.000	-	-	-
logvBS	1.000	0.000	-	-	-
Iterations[a]	5000				
PSR[b]	1.085				
DIC	287612.031				

Notes. *N* = 79. Number of observations = 3'063. All parameters in this table are standardized. APS = Attenuated psychotic symptoms. BS = basic symptoms. LCI = lower part of confidence interval. UCI = upper part of confidence interval. DIC = Deviance Information Criterion. PSR = Potential Scale Reduction. 'A → B' symbolizes a directional effect of A on B (i.e., B regressed on A). 'A ←→ B' symbolizes an undirectional covariation between A and B.
[a] The minimum number of iterations was set at 5000. The model converged at 5900 iterations.
[b] Value of the last iteration.
[c] P-value is one-tailed. Values below 0.025 are considered significant.
[d] The random residual variance (v) of normally distributed random effects has a lognormal distribution and is therefore called 'logv'.

<u>Commentary</u>

Among the cross-lagged, non-random paths, the effect of APS/B(L)IPS$_{t-1}$ on BS$_t$ (st.est: 0.009) was not significant, whereas the effect of BS$_{t-1}$ on APS/B(L)IPS$_t$ (st.est: 0.273) was significant. The random autoregressive effects of BS (st.est.: 0.567), APS/B(L)IPS (st.est: 0.217), and stress (st.est: 0.389) were significant, with BS having a significantly greater autoregressive effect compared to APS/B(L)IPS. The lag0-effects of APS/B(L)IPS$_t$ on stress$_t$ (st.est: 0.156) and of BS$_t$ on stress$_t$ (st.est: 0.136) were both significant and of similar magnitude (i.e., overlapping CIs). The between level APS/B(L)IPS and BS scores showed a high, significant correlation (0.885).

Full report of the model with CHR symptoms split into perceptive versus non-perceptive symptoms

Table 9 of SM

Results of Model C with CHR symptoms split into perceptive (PERC) and non-perceptive symptoms (NONP)

**Model A**						
Within-Level standardized estimates averaged across clusters		Estimate	SD	P^c	LCI	UCI
**Effects**						
PERC (T-1) → Stress (T)		0.011	0.015	0.225	-0.016	0.045
NONP (T-1) → Stress (T)		0.029	0.020	0.079	-0.009	0.066
NONP (T-1) → PERC (T)		0.046*	0.017	0.002	0.013	0.079
Stress (T-1) → PERC (T)		-0.007	0.016	0.346	-0.037	0.024
PERC (T-1) → NONP (T)		-0.082*	0.012	0.000	-0.110	-0.063
Stress (T-1) → NONP (T)		0.010	0.007	0.070	-0.004	0.024
**Effects with random slopes (rs)**						
rCov	PERC ←→ NONP	0.435*	0.017	0.000	0.401	0.467
rsStress:	Stress (T-1) → Stress (T)	0.384*	0.026	0.000	0.335	0.435
rsPERC:	PERC (T-1) → PERC (T)	0.191*	0.030	0.000	0.134	0.251
rsNONP:	NONP (T-1) → NONP (T)	0.564*	0.021	0.000	0.522	0.602
rsPERCLag0:	PERC (T) → Stress (T)	0.062*	0.024	0.010	0.011	0.106
rsNONPLag0:	NONP (T) → Stress (T)	0.196*	0.024	0.000	0.149	0.239
**Random residual variance**[d]						
logvStress:	logv for Stress	0.663*	0.018	0.000	0.627	0.699
logvPERC:	logv for PERC	0.855*	0.014	0.000	0.828	0.882
logvNONP:	logv for NONP	0.663*	0.017	0.000	0.628	0.697
**R^2**						
Stress		0.337*	0.018	0.000	0.301	0.373
PERC		0.145*	0.014	0.000	0.117	0.172
NONP		0.337*	0.017	0.000	0.303	0.372
Between Level		Estimate	SD	P^c	LCI	UCI
**Covariations**						
Stress ←→						
	rsStress	0.144	0.171	0.214	-0.204	0.452
	rsPERC	-0.109	0.173	0.272	-0.425	0.244
	rsNONP	-0.212	0.160	0.105	-0.502	0.113
	rsPERCLag0	-0.064	0.171	0.362	-0.389	0.271
	rsNONPLag0	0.160	0.161	0.167	-0.166	0.460

	rCov	0.002	0.151	0.494	-0.282	0.297
	logvStress	0.316*	0.130	0.012	0.035	0.540
	logvPERC	0.071	0.137	0.307	-0.196	0.335
	logvNONP	0.135	0.134	0.173	-0.136	0.383
PERC ←→						
	rsStress	-0.356	0.185	0.026	-0.708	0.002
	rsPERC	0.135	0.159	0.206	-0.186	0.438
	rsNONP	0.064	0.161	0.353	-0.260	0.360
	rsPERCLag0	0.224	0.180	0.119	-0.156	0.542
	rsNONPLag0	-0.310	0.154	0.036	-0.572	0.030
	rCov	0.075	0.145	0.307	-0.209	0.350
	logvStress	0.103	0.138	0.230	-0.180	0.362
	logvPERC	0.448*	0.111	0.000	0.208	0.635
	logvNONP	0.418*	0.112	0.000	0.175	0.617
NONP ←→						
	rsStress	-0.262	0.182	0.082	-0.606	0.103
	rsPERC	0.079	0.163	0.320	-0.245	0.395
	rsNONP	-0.064	0.164	0.358	-0.383	0.247
	rsPERCLag0	0.151	0.196	0.242	-0.262	0.489
	rsNONPLag0	-0.222	0.172	0.119	-0.516	0.158
	rCov	0.003	0.150	0.491	-0.286	0.298
	logvStress	0.182	0.137	0.105	-0.105	0.428
	logvPERC	0.411*	0.115	0.001	0.168	0.618
	logvNONP	0.548*	0.097	0.000	0.326	0.710
rsStress ←→						
	rsPERC	0.194	0.194	0.172	-0.201	0.549
	rsNONP	0.308	0.190	0.067	-0.096	0.629
	rsPERCLag0	0.215	0.229	0.183	-0.224	0.654
	rsNONPLag0	-0.054	0.211	0.398	-0.468	0.352
	rCov	0.052	0.181	0.383	-0.311	0.402
	logvStress	0.040	0.213	0.434	-0.367	0.449
	logvPERC	-0.294	0.161	0.047	-0.572	0.051
	logvNONP	-0.060	0.172	0.366	-0.377	0.293
rsPERC ←→						
	rsNONP	0.454*	0.177	0.011	0.073	0.759
	rsPERCLag0	0.065	0.220	0.392	-0.375	0.485
	rsNONPLag0	-0.028	0.193	0.446	-0.398	0.351
	rCov	0.276	0.163	0.054	-0.069	0.563
	logvStress	-0.197	0.164	0.127	-0.504	0.144
	logvPERC	0.262	0.160	0.062	-0.081	0.546
	logvNONP	0.135	0.159	0.198	-0.182	0.436
rsNONP ←→						

	rsPERCLag0	0.261	0.200	0.110	-0.185	0.599
	rsNONPLag0	-0.220	0.185	0.131	-0.556	0.170
	rCov	-0.085	0.177	0.314	-0.417	0.266
	logvStress	-0.076	0.159	0.316	-0.383	0.235
	logvPERC	-0.037	0.169	0.410	-0.368	0.292
	logvNONP	-0.085	0.163	0.298	-0.401	0.246
rsPERCLag0 ←→						
	rsNONPLag0	-0.603*	0.205	0.010	-0.901	-0.102
	rCov	0.227	0.204	0.147	-0.218	0.576
	logvStress	-0.074	0.157	0.324	-0.369	0.236
	logvPERC	0.106	0.260	0.358	-0.417	0.558
	logvNONP	0.323	0.259	0.151	-0.271	0.701
rsNONPLag0 ←→						
	rCov	-0.175	0.164	0.153	-0.470	0.167
	logvStress	0.247	0.152	0.059	-0.069	0.530
	logvPERC	-0.181	0.197	0.198	-0.517	0.239
	logvNONP	-0.424	0.219	0.056	-0.740	0.111
	logvStress	-0.163	0.146	0.147	-0.434	0.132
	logvPERC	0.585*	0.103	0.000	0.345	0.751
	logvNONP	0.455*	0.122	0.001	0.190	0.660
	logvPERC	0.135	0.136	0.168	-0.142	0.393
	logvNONP	0.175	0.135	0.099	-0.102	0.425
logvPERC ←→						
	logvNONP	0.710*	0.072	0.000	0.546	0.825
Stress ←→						
	PERC	0.205	0.133	0.075	-0.073	0.441
	NONP	0.401*	0.117	0.001	0.145	0.602
PERC ←→						
	NONP	0.780*	0.059	0.000	0.638	0.866
**Means**						
	Stress	1.436*	0.181	0.000	1.101	1.817
	PERC	0.690*	0.133	0.000	0.428	0.954
	NONP	1.050*	0.154	0.000	0.759	1.359
	rsStress	1.339*	0.273	0.000	0.880	1.941
	rsPERC	0.528*	0.173	0.000	0.226	0.898
	rsNONP	2.181*	0.382	0.000	1.533	3.020
	rsPERCLag0	0.126	0.217	0.282	-0.266	0.561
	rsNONPLag0	0.819*	0.188	0.000	0.471	1.210
	rCov	1.056*	0.170	0.000	0.740	1.402

logvStress	4.324*	0.463	0.000	3.472	5.291
logvPERC	1.283*	0.170	0.000	0.961	1.629
logvNONP	1.713*	0.203	0.000	1.325	2.124
**Residual Variances**					
Stress	1.000	0.000	-	-	-
PERC	1.000	0.000	-	-	-
NONP	1.000	0.000	-	-	-
rsStress	1.000	0.000	-	-	-
rsPERC	1.000	0.000	-	-	-
rsNONP	1.000	0.000	-	-	-
rsPERCLag0	1.000	0.000	-	-	-
rsNONPLag0	1.000	0.000	-	-	-
rCov	1.000	0.000			
logvStress	1.000	0.000	-	-	-
logvPERC	1.000	0.000	-	-	-
logvNONP	1.000	0.000	-	-	-
Iterations[a]	5000				
PSR[b]	1.039				
DIC	283214.309				

Notes. N = 79. Number of observations = 3'063. All parameters in this table are standardized. PERC = perceptive psychotic symptoms. NONP = non-perceptive psychotic symptoms. LCI = lower part of confidence interval. UCI = upper part of confidence interval. DIC = Deviance Information Criterion. PSR = Potential Scale Reduction. 'A → B' symbolizes a directional effect of A on B (i.e., B regressed on A). 'A ←→ B' symbolizes an undirectional covariation between A and B.

[a] The minimum number of iterations was set at 5000. The model converged at this minimum.

[b] Value of the last iteration.

[c] P-value is one-tailed. Values below 0.025 are considered significant.

[d] The random residual variance (v) of normally distributed random effects has a lognormal distribution and is therefore called 'logv'.

Commentary

The cross-lagged, non-random paths of $NONP_{t-1}$ on $PERC_t$ (st.est: 0.046) and $PERC_{t-1}$ on $NONP_t$ (-0.082) were significant. Notably, the latter effect changed sign after introducing random covariation between NONP and PERC into the model. The random slopes of the autoregressive paths for PERC (st.est: 0.191), NONP (st.est: 0.564), and stress (st.est: 0.384) were significant, with NONP having a significantly greater autoregressive effect compared to PERC (i.e., CIs not overlapping). The lag0-effects of $PERC_t$ on $stress_t$ (st.est: 0.062) and $NONP_t$ on $stress_t$ (st.est: 0.196) were both significant, with the latter being of significantly higher magnitude (i.e., CIs not overlapping). The between level PERC and NONP scores showed a high, significant correlation (0.780).

Mplus code for conducting R-DSEM

Model A

```
DATA:
      FILE = "T:\pub_2023_fetz-ema-stress\data\processed\data_mplus_reduced.dat";
VARIABLE:
      NAMES = id timept str bs perc nperc aps pe mood hours hours_2 hours_3 hours_or sex
               age;
      MISSING=.;
      USEVARIABLES = str pe hours_or;
      CLUSTER = id;
      LAGGED = str(1) pe(1);
      TINTERVAL = hours_or (1 time);

ANALYSIS:
      TYPE = TWOLEVEL RANDOM;
      ESTIMATOR = BAYES;
      PROCESSORS = 10;
      BITERATIONS = (2000);
      THIN= 10;

MODEL:
            %WITHIN%
            str^ ON str^1 pe^1;
            pe^ ON pe^1 str^1;
            str WITH pe !covar
            %BETWEEN%
            str WITH pe;

OUTPUT:
      standardized tech1 tech4 tech8;

PLOT:
```

```
        type = plot3;
```

Model B

```
 DATA:
        FILE = "T:\pub_2023_fetz-ema-stress\data\processed\data_mplus_reduced.dat";
 VARIABLE:
        NAMES = id timept str bs perc nperc aps pe mood hours hours_2 hours_3 hours_or sex
              age;
        MISSING=.;
        USEVARIABLES = str pe hours_or;
        CLUSTER = id;
        LAGGED = str(1) pe(1);
        TINTERVAL = hours_or (1 time);

 ANALYSIS:
       TYPE = TWOLEVEL RANDOM;
       ESTIMATOR = BAYES;
       PROCESSORS = 10;
       BITERATIONS = (2000);
       THIN= 10;

 MODEL:
              %WITHIN%
              str^ ON str^1 pe^1;
              pe^ ON pe^1 str^1;
              str ON pe !lag0 pe --> stress
              %BETWEEN%
              str WITH pe;

 OUTPUT:
       standardized tech1 tech4 tech8;
```

```
  PLOT:
        type = plot3;

!END
```

Model C

```
DATA:
        FILE = "T:\pub_2023_fetz-ema-stress\data\processed\data_mplus_reduced.dat";
VARIABLE:
        NAMES = id timept str bs perc nperc aps pe mood hours hours_2 hours_3 hours_or sex
                age;
        MISSING=.;
        USEVARIABLES = str pe hours_or;
        CLUSTER = id;
        LAGGED = str(1) pe(1); !The LAGGED option triggers time series analysis
        TINTERVAL = hours_or (1 time);

ANALYSIS:
       TYPE = TWOLEVEL RANDOM;
       ESTIMATOR = BAYES;
       PROCESSORS = 10;
       BITERATIONS = (2000);
       THIN= 10;

MODEL:
            %WITHIN%
            str^ ON str^1 pe^1;
            pe^ ON pe^1 str^1;
            pe ON str !lag0 for stress --> pe

            %BETWEEN%
            str WITH pe;

OUTPUT:
       standardized tech1 tech4 tech8;

PLOT:
```

type = plot3;

!END

Model C with random effects

```
 DATA:
        FILE = "T:\pub_2023_fetz-ema-stress\data\processed\data_mplus_reduced.dat";
 VARIABLE:
        NAMES = id timept str bs perc nperc aps pe mood hours hours_2 hours_3 hours_or
sex
               age;
        MISSING=.;
        USEVARIABLES = str pe hours_or;
        CLUSTER = id;
        LAGGED = str(1) pe(1); !The LAGGED option triggers time series analysis
        TINTERVAL = hours_or (1 time);

 ANALYSIS:
       TYPE = TWOLEVEL RANDOM;
       ESTIMATOR = BAYES;
       PROCESSORS = 10;
       BITERATIONS = (2000);
       THIN= 10;

 MODEL:
              %WITHIN%
              rs | str^ ON str^1;
              str^ ON pe^1;
              rp | pe^ ON pe^1;
              pe^ ON str^1;
              slag0 | str ON pe; !LAG0
              logvs | str;
              logvp | pe;

              %BETWEEN%
              str pe rs-logvp  WITH str pe rs-logvp;
```

```
 OUTPUT:
        standardized tech1 tech4 tech8;

 PLOT:
        type = plot3;

!END
```

Model C with regression of random effects on moderators

```
DATA: FILE = "T:/pub_2023_fetz-ema-stress/data/processed/data_mplus_reduced_mod.dat";
 VARIABLE:
       NAMES = id timept str bs perc nperc aps pe mood hours hours_2 hours_3 hours_or sex
                    age mcomp sev_bs sev_aps;
       MISSING=.;
       USEVARIABLES = str pe hours_or sex age sev_bs sev_aps;
       CLUSTER = id;
       LAGGED = str(1) pe(1);
       TINTERVAL = hours_or (1 time);
       BETWEEN= sex age sev_bs sev_aps;

       USEOBSERVATIONS = id ne 118; !excluded, no diagnostics data (clinical interviews)

 ANALYSIS:
      TYPE = TWOLEVEL RANDOM;
      ESTIMATOR = BAYES;
      PROCESSORS = 10;
      BITERATIONS = (2000);
      THIN= 10;

 MODEL:
            %WITHIN%
            rs | str^ ON str^1;
            str^ ON pe^1;
            rp | pe^ ON pe^1;
            pe^ ON str^1;
            slag0 | str ON pe; !LAG0
            logvs | str;
            logvp | pe;
```

```
%BETWEEN%
str pe rs-logvp  WITH str pe rs-logvp;
str pe rs-logvp ON age sex sev_bs sev_aps; !regression of random effects on moderators/covariates

[logvs] (mst);
[logvp] (mpe);
logvs (sst);
logvp (spe);

MODEL CONSTRAINT:
NEW (meanvst meanvpe);
meanvst = exp(mst+sst/2);
meanvpe = exp(mpe+spe/2);

OUTPUT:
standardized tech1 tech4 tech8;

PLOT:
type = plot3;

!END
```

Model C with joint testing of the moderators (Wald Test)

```
DATA: FILE = "T:/pub_2023_fetz-ema-stress/data/processed/data_mplus_reduced_mod.dat";
VARIABLE:
NAMES = id timept str bs perc nperc aps pe mood hours hours_2 hours_3 hours_or sex
age mcomp sev_bs sev_aps;
MISSING=.;
USEVARIABLES = str pe hours_or sex age sev_bs sev_aps; !CHECK
CLUSTER = id;
LAGGED = str(1) pe(1);
```

```
      TINTERVAL = hours_or (1 time);
      BETWEEN= sex age sev_bs sev_aps;

ANALYSIS:
     TYPE = TWOLEVEL RANDOM;
     ESTIMATOR = BAYES;
     PROCESSORS = 10;
     BITERATIONS = (2000);
     THIN= 10;

MODEL:
          %WITHIN%
          rs | str^ ON str^1;
          str^ ON pe^1;
          rp | pe^ ON pe^1;
          pe^ ON str^1;
          slag0 | str ON pe; !LAG0
          logvs | str;
          logvp | pe;

          %BETWEEN%
          str pe rs-logvp  WITH str pe rs-logvp;
          slag0 ON age sex sev_bs sev_aps (m1-m4);

          [logvs] (mst);
          [logvp] (mpe);
          logvs (sst);
          logvp (spe);

MODEL CONSTRAINT:
          NEW (meanvst meanvpe);
          meanvst = exp(mst+sst/2);
          meanvpe = exp(mpe+spe/2);
```

```
MODEL TEST:
        m1 = 0;
        m2 = 0;
        m3 = 0;
        m4 = 0;

OUTPUT:
    standardized tech1 tech4 tech8;

PLOT:
    type = plot3;

!END
```

<u>Model C with CHR split into APS/B(L)IPS and BS (analogous to perceptive versus non-perceptive symptoms)</u>

```
TITLE:          str_pe_cov_sex_age_DSEM_no_RE
DATA:
     FILE = "T:\pub_2023_fetz-ema-stress\data\processed\data_mplus_reduced.dat";
VARIABLE:
     NAMES = id timept str bs perc nperc aps pe mood hours hours_2 hours_3 hours_or
sex
          age;
     MISSING=.;
     USEVARIABLES = str aps bs hours_or;
     CLUSTER = id;
     LAGGED = str(1) aps(1) bs(1);
     TINTERVAL = hours_or (1 time);

ANALYSIS:
    TYPE = TWOLEVEL RANDOM;
```

```
        ESTIMATOR = BAYES;
        PROCESSORS = 10;
        BITERATIONS = (5000);
        !THIN= 10;

  MODEL:
                %WITHIN%
                rs | str^ ON str^1;
                str^ ON aps^1 bs^1;
                ra | aps^ ON aps^1;
                aps^ ON bs^1 str^1;
                rb | bs^ ON bs^1;
                bs^ ON aps^1 str^1;
                slag0a | str ON aps; !lag0 for aps
                slag0b | str ON bs; !lag0 for bs
                cov | aps WITH bs; !added during 1. revision (random covariation)
                logvs | str;
                logva | aps;
                logvb | bs;

                %BETWEEN%
                str aps bs rs-logvb WITH str aps bs rs-logvb;



  OUTPUT:
        standardized tech1 tech4 tech8;

  PLOT:
        type = plot3;

!END
```

Evaluating model structures with simulated data from a lag0 model structure without cross-lagged effects (Monte Carlo Simulation)

Fitting true model

```
Mplus VERSION 8.10
MUTHEN & MUTHEN
09/12/2024   1:52 PM

INPUT INSTRUCTIONS

  TITLE:
  dikj
  MONTECARLO:
  NAMES ARE y1-y2 ;
  NOBSERVATIONS = 25000;
  NREPS = 100;
    NCSIZES = 1; CSIZES = 250(100);
    lagged = y1(1) y2(1);
    SEED = 14;
  MODEL POPULATION:

  %WITHIN%

  p1 | y2 ON y1;
  y1^ ON y1^1@0.6;
  y2^ ON y2^1@0.4;
  p2 | y1;
  p3 | y2;


   %BETWEEN%
   [p1*0.5 p2*0 p3*0];
  p1*0.0625 p2*0.25 p3*0.25;


  y1@3;
  y2@3;
  ANALYSIS:
  TYPE IS TWOLEVEL RANDOM;
    estimator=bayes; proc=8;
    BSEED = 23;
    FBITER = 400;
  MODEL:

   %WITHIN%

  y2 ON y1*0.5;
  y1^ ON y1^1*0.6;
   y2^ ON y2^1*0.4;
  y2^ ON y1^1*0;
  y1^ ON y2^1*0;
  y1*1.14;
  y2*1.14;


   %BETWEEN%

  y1 WITH y2*0;
```

```
  OUTPUT:
  Standardized;



*** WARNING in OUTPUT command
  STANDARDIZED option is not available for MONTECARLO.
  Request for STANDARDIZED is ignored.
   1 WARNING(S) FOUND IN THE INPUT INSTRUCTIONS




dikj

SUMMARY OF ANALYSIS

Number of groups                                                 1
Number of observations                                       25000

Number of replications
    Requested                                                  100
    Completed                                                  100
Value of seed                                                   14

Number of dependent variables                                    2
Number of independent variables                                  2
Number of continuous latent variables                            0

Observed dependent variables

  Continuous
   Y1          Y2

Observed independent variables
   Y1^1        Y2^1

Variables with special functions

  Within variables
   Y1^1        Y2^1


Estimator                                                    BAYES
Specifications for Bayesian Estimation
  Point estimate                                            MEDIAN
  Number of Markov chain Monte Carlo (MCMC) chains               2
  Random seed for the first chain                               23
  Starting value information                           UNPERTURBED
  Algorithm used for Markov chain Monte Carlo           GIBBS(PX1)
  Fixed number of iterations                                   400
  K-th iteration used for thinning                               1


SUMMARY OF DATA FOR THE FIRST REPLICATION

     Cluster information

       Size (s)    Number of clusters of Size s

```

```
        100           250




MODEL FIT INFORMATION

Number of Free Parameters                         12

Information Criteria

    Deviance (DIC)

        Mean                              150372.973
        Std Dev                             1200.624
        Number of successful computations      100

             Proportions                   Percentiles
        Expected    Observed         Expected       Observed
           0.990       0.990       147579.962     147530.101
           0.980       0.980       147907.252     147591.964
           0.950       0.930       148398.067     148217.765
           0.900       0.880       148834.253     148633.786
           0.800       0.820       149362.528     149395.569
           0.700       0.720       149743.366     149766.468
           0.500       0.460       150372.973     150277.860
           0.300       0.330       151002.580     151018.619
           0.200       0.220       151383.417     151506.967
           0.100       0.140       151911.692     152091.494
           0.050       0.060       152347.878     152352.192
           0.020       0.000       152838.693     152460.231
           0.010       0.000       153165.983     152509.537

    Estimated Number of Parameters (pD)

        Mean                                 497.602
        Std Dev                                6.642
        Number of successful computations      100

             Proportions                   Percentiles
        Expected    Observed         Expected       Observed
           0.990       0.990          482.150        482.002
           0.980       0.960          483.961        483.103
           0.950       0.950          486.676        485.798
           0.900       0.900          489.089        488.828
           0.800       0.770          492.012        491.450
           0.700       0.720          494.119        494.259
           0.500       0.460          497.602        496.790
           0.300       0.320          501.085        501.413
           0.200       0.270          503.192        503.692
           0.100       0.120          506.115        506.749
           0.050       0.050          508.528        508.340
           0.020       0.000          511.244        510.453
           0.010       0.000          513.054        510.465



MODEL RESULTS
```

```
                                ESTIMATES              S. E.     M. S. E.
95%  % Sig
                  Population   Average   Std. Dev.   Average          Cover
Coeff

Within Level

 Y2          ON
  Y1                   0.500      0.5031      0.0406      0.0130      0.0016
0.460 1.000

 Y1^         ON
  Y1^1                 0.600      0.5995      0.0059      0.0053      0.0000
0.900 1.000
  Y2^1                 0.000      0.0008      0.0054      0.0056      0.0000
0.930 0.070

 Y2^         ON
  Y2^1                 0.400      0.4146      0.0069      0.0059      0.0003
0.330 1.000
  Y1^1                 0.000     -0.0007      0.0077      0.0087      0.0001
0.980 0.020

 Residual Variances
  Y1                   1.140      1.1310      0.0420      0.0102      0.0018
0.400 1.000
  Y2                   1.140      1.2094      0.0358      0.0110      0.0061
0.090 1.000

Between Level

 Y1        WITH
  Y2                   0.000      0.0045      0.1994      0.2004      0.0394
0.950 0.050

 Means
  Y1                   0.000     -0.0037      0.1164      0.1127      0.0134
0.950 0.050
  Y2                   0.000     -0.0019      0.1172      0.1105      0.0136
0.940 0.060

 Variances
  Y1                   0.500      3.0779      0.2723      0.2877      6.7192
0.000 1.000
  Y2                   0.500      2.9824      0.2591      0.2792      6.2290
0.000 1.000


CORRELATIONS AND MEAN SQUARE ERROR OF THE TRUE FACTOR VALUES AND THE
FACTOR SCORES

                        CORRELATIONS            MEAN SQUARE ERROR
                    Average     Std. Dev.          Average     Std. Dev.
  Y1                 -0.013       0.061             1.809       0.079
  Y2                  0.006       0.065             1.763       0.081


TECHNICAL 1 OUTPUT
```

```
     PARAMETER SPECIFICATION FOR WITHIN


           NU
              Y1            Y2            Y1^1          Y2^1
              ________      ________      ________      ________
                    0             0             0             0


           LAMBDA
              Y1            Y2            Y1^1          Y2^1
              ________      ________      ________      ________
 Y1                 0             0             0             0
 Y2                 0             0             0             0
 Y1^1               0             0             0             0
 Y2^1               0             0             0             0


           THETA
              Y1            Y2            Y1^1          Y2^1
              ________      ________      ________      ________
 Y1                 0
 Y2                 0             0
 Y1^1               0             0             0
 Y2^1               0             0             0             0


           ALPHA
              Y1            Y2            Y1^1          Y2^1
              ________      ________      ________      ________
                    0             0             0             0


           BETA
              Y1            Y2            Y1^1          Y2^1
              ________      ________      ________      ________
 Y1                 0             0             1             2
 Y2                 3             0             4             5
 Y1^1               0             0             0             0
 Y2^1               0             0             0             0


           PSI
              Y1            Y2            Y1^1          Y2^1
              ________      ________      ________      ________
 Y1                 6
 Y2                 0             7
 Y1^1               0             0             0
 Y2^1               0             0             0             0


     PARAMETER SPECIFICATION FOR BETWEEN


           NU
              Y1            Y2
              ________      ________
                    0             0
```

```
           LAMBDA
              Y1            Y2
              ________      ________
 Y1                 0             0
 Y2                 0             0


           THETA
              Y1            Y2
              ________      ________
 Y1                 0
 Y2                 0             0


           ALPHA
              Y1            Y2
              ________      ________
                    8             9


           BETA
              Y1            Y2
              ________      ________
 Y1                 0             0
 Y2                 0             0


           PSI
              Y1            Y2
              ________      ________
 Y1                10
 Y2                11            12


     STARTING VALUES FOR WITHIN


           NU
              Y1            Y2            Y1^1          Y2^1
              ________      ________      ________      ________
                0.000         0.000         0.000         0.000


           LAMBDA
              Y1            Y2            Y1^1          Y2^1
              ________      ________      ________      ________
 Y1             1.000         0.000         0.000         0.000
 Y2             0.000         1.000         0.000         0.000
 Y1^1           0.000         0.000         1.000         0.000
 Y2^1           0.000         0.000         0.000         1.000


           THETA
              Y1            Y2            Y1^1          Y2^1
              ________      ________      ________      ________
 Y1             0.000
 Y2             0.000         0.000
 Y1^1           0.000         0.000         0.000
 Y2^1           0.000         0.000         0.000         0.000
```

```
           ALPHA
              Y1            Y2            Y1^1          Y2^1
              ________      ________      ________      ________
                0.000         0.000         0.000         0.000


           BETA
              Y1            Y2            Y1^1          Y2^1
              ________      ________      ________      ________
 Y1             0.000         0.000         0.600         0.000
 Y2             0.500         0.000         0.000         0.400
 Y1^1           0.000         0.000         0.000         0.000
 Y2^1           0.000         0.000         0.000         0.000


           PSI
              Y1            Y2            Y1^1          Y2^1
              ________      ________      ________      ________
 Y1             1.140
 Y2             0.000         1.140
 Y1^1           0.000         0.000         0.500
 Y2^1           0.000         0.000         0.000         0.500


     STARTING VALUES FOR BETWEEN


           NU
              Y1            Y2
              ________      ________
                0.000         0.000


           LAMBDA
              Y1            Y2
              ________      ________
 Y1             1.000         0.000
 Y2             0.000         1.000


           THETA
              Y1            Y2
              ________      ________
 Y1             0.000
 Y2             0.000         0.000


           ALPHA
              Y1            Y2
              ________      ________
                0.000         0.000


           BETA
              Y1            Y2
              ________      ________
 Y1             0.000         0.000
 Y2             0.000         0.000
```

```
           PSI
              Y1            Y2
              ________      ________
 Y1             0.500
 Y2             0.000         0.500


     POPULATION VALUES FOR WITHIN


           NU
              Y1            Y2            Y1^1          Y2^1
              ________      ________      ________      ________
                0.000         0.000         0.000         0.000


           LAMBDA
              Y1            Y2            Y1^1          Y2^1
              ________      ________      ________      ________
 Y1             1.000         0.000         0.000         0.000
 Y2             0.000         1.000         0.000         0.000
 Y1^1           0.000         0.000         1.000         0.000
 Y2^1           0.000         0.000         0.000         1.000


           THETA
              Y1            Y2            Y1^1          Y2^1
              ________      ________      ________      ________
 Y1             0.000
 Y2             0.000         0.000
 Y1^1           0.000         0.000         0.000
 Y2^1           0.000         0.000         0.000         0.000


           ALPHA
              Y1            Y2            Y1^1          Y2^1
              ________      ________      ________      ________
                0.000         0.000         0.000         0.000


           BETA
              Y1            Y2            Y1^1          Y2^1
              ________      ________      ________      ________
 Y1             0.000         0.000         0.600         0.000
 Y2             0.000         0.000         0.000         0.400
 Y1^1           0.000         0.000         0.000         0.000
 Y2^1           0.000         0.000         0.000         0.000


           PSI
              Y1            Y2            Y1^1          Y2^1
              ________      ________      ________      ________
 Y1             0.000
 Y2             0.000         0.000
 Y1^1           0.000         0.000         1.000
 Y2^1           0.000         0.000         0.000         1.000


     POPULATION VALUES FOR BETWEEN
```

NU

Y1	Y2
0.000	0.000

LAMBDA

	P1	P2	P3	Y1	Y2
Y1	0.000	0.000	0.000	1.000	0.000
Y2	0.000	0.000	0.000	0.000	1.000

THETA

	Y1	Y2
Y1	0.000	
Y2	0.000	0.000

ALPHA

P1	P2	P3	Y1	Y2
0.500	0.000	0.000	0.000	0.000

BETA

	P1	P2	P3	Y1	Y2
P1	0.000	0.000	0.000	0.000	0.000
P2	0.000	0.000	0.000	0.000	0.000
P3	0.000	0.000	0.000	0.000	0.000
Y1	0.000	0.000	0.000	0.000	0.000
Y2	0.000	0.000	0.000	0.000	0.000

PSI

	P1	P2	P3	Y1	Y2
P1	0.062				
P2	0.000	0.250			
P3	0.000	0.000	0.250		
Y1	0.000	0.000	0.000	3.000	
Y2	0.000	0.000	0.000	0.000	3.000

PRIORS FOR ALL PARAMETERS	PRIOR MEAN	PRIOR VARIANCE	PRIOR STD. DEV.
Parameter 1~N(0.000,infinity)	0.0000	infinity	infinity
Parameter 2~N(0.000,infinity)	0.0000	infinity	infinity

```
     Parameter 3~N(0.000,infinity)           0.0000            infinity
infinity
     Parameter 4~N(0.000,infinity)           0.0000            infinity
infinity
     Parameter 5~N(0.000,infinity)           0.0000            infinity
infinity
     Parameter 6~IG(-1.000,0.000)          infinity            infinity
infinity
     Parameter 7~IG(-1.000,0.000)          infinity            infinity
infinity
     Parameter 8~N(0.000,infinity)           0.0000            infinity
infinity
     Parameter 9~N(0.000,infinity)           0.0000            infinity
infinity
     Parameter 10~IW(0.000,-3)             infinity            infinity
infinity
     Parameter 11~IW(0.000,-3)             infinity            infinity
infinity
     Parameter 12~IW(0.000,-3)             infinity            infinity
infinity


DIAGRAM INFORMATION

  Mplus diagrams are currently not available for multilevel analysis.
  No diagram output was produced.


     Beginning Time:  13:52:17
        Ending Time:  14:01:17
       Elapsed Time:  00:09:00



MUTHEN & MUTHEN
3463 Stoner Ave.
Los Angeles, CA  90066

Tel: (310) 391-9971
Fax: (310) 391-8971
Web: www.StatModel.com
Support: Support@StatModel.com

Copyright (c) 1998-2023 Muthen & Muthen
```

Fitting covariance between residuals (instead of lag0)

```
Mplus VERSION 8.10
MUTHEN & MUTHEN
09/12/2024   3:36 PM

INPUT INSTRUCTIONS

  TITLE:
  dikj
  MONTECARLO:
  NAMES ARE y1-y2 ;
  NOBSERVATIONS = 25000;
  NREPS = 100;
    NCSIZES = 1; CSIZES = 250(100);
    lagged = y1(1) y2(1);
    SEED = 14;
  MODEL POPULATION:

  %WITHIN%

  p1 | y1 ON y2;
  y1^ ON y1^1@0.6;
  y2^ ON y2^1@0.4;
  p2 | y1;
  p3 | y2;


   %BETWEEN%
   [p1*0.5 p2*0 p3*0];
  p1*0.0625 p2*0.25 p3*0.25;


  y1@3;
  y2@3;
  ANALYSIS:
  TYPE IS TWOLEVEL RANDOM;
    estimator=bayes; proc=8;
    BSEED = 23;
    FBITER = 400;
  MODEL:

   %WITHIN%

  y2 WITH y1*0.5;
  y1^ ON y1^1*0.6;
   y2^ ON y2^1*0.4;
  y2^ ON y1^1*0;
  y1^ ON y2^1*0;
  y1*1.14;
  y2*1.14;


   %BETWEEN%

  y1 WITH y2*0;
  OUTPUT:
  Standardized;
```

```
*** WARNING in OUTPUT command
  STANDARDIZED option is not available for MONTECARLO.
  Request for STANDARDIZED is ignored.
   1 WARNING(S) FOUND IN THE INPUT INSTRUCTIONS




dikj

SUMMARY OF ANALYSIS

Number of groups                                                 1
Number of observations                                       25000

Number of replications
    Requested                                                  100
    Completed                                                  100
Value of seed                                                   14

Number of dependent variables                                    2
Number of independent variables                                  2
Number of continuous latent variables                            0

Observed dependent variables

  Continuous
   Y1          Y2

Observed independent variables
   Y1^1        Y2^1

Variables with special functions

  Within variables
   Y1^1        Y2^1


Estimator                                                    BAYES
Specifications for Bayesian Estimation
  Point estimate                                            MEDIAN
  Number of Markov chain Monte Carlo (MCMC) chains                 2
  Random seed for the first chain                                 23
  Starting value information                           UNPERTURBED
  Algorithm used for Markov chain Monte Carlo           GIBBS(PX1)
  Fixed number of iterations                                     400
  K-th iteration used for thinning                                 1


SUMMARY OF DATA FOR THE FIRST REPLICATION

     Cluster information

       Size (s)    Number of clusters of Size s

        100           250
```

MODEL FIT INFORMATION

Number of Free Parameters 12

Information Criteria

Deviance (DIC)

Mean	150314.421
Std Dev	1178.180
Number of successful computations	100

Proportions		Percentiles	
Expected	Observed	Expected	Observed
0.990	0.980	147573.621	147419.999
0.980	0.980	147894.793	147433.389
0.950	0.930	148376.433	148222.301
0.900	0.890	148804.466	148479.363
0.800	0.830	149322.865	149437.446
0.700	0.720	149696.584	149748.405
0.500	0.480	150314.421	150235.922
0.300	0.320	150932.259	150987.306
0.200	0.230	151305.978	151379.819
0.100	0.120	151824.377	152004.540
0.050	0.050	152252.410	152225.376
0.020	0.000	152734.050	152415.383
0.010	0.000	153055.222	152468.675

Estimated Number of Parameters (pD)

Mean	491.585
Std Dev	4.666
Number of successful computations	100

Proportions		Percentiles	
Expected	Observed	Expected	Observed
0.990	0.990	480.730	478.509
0.980	0.980	482.002	480.834
0.950	0.930	483.910	483.486
0.900	0.860	485.605	484.691
0.800	0.830	487.658	487.889
0.700	0.700	489.138	488.913
0.500	0.500	491.585	491.576
0.300	0.290	494.032	493.970
0.200	0.190	495.512	495.211
0.100	0.100	497.565	497.527
0.050	0.050	499.260	498.896
0.020	0.010	501.167	499.633
0.010	0.010	502.439	500.175

MODEL RESULTS

ESTIMATES S. E. M. S. E.
95% % Sig

```
                     Population   Average   Std. Dev.   Average          Cover
Coeff

Within Level

 Y1^        ON
  Y1^1                 0.600     0.5896     0.0070     0.0059     0.0002
0.550 1.000
  Y2^1                 0.000    -0.0938     0.0087     0.0074     0.0089
0.000 1.000

 Y2^        ON
  Y2^1                 0.400     0.4001     0.0076     0.0065     0.0001
0.890 1.000
  Y1^1                 0.000    -0.0007     0.0052     0.0051     0.0000
0.950 0.050

 Y2       WITH
  Y1                   0.500     0.5704     0.0285     0.0091     0.0058
0.040 1.000

 Residual Variances
  Y1                   1.140     1.4904     0.0473     0.0135     0.1250
0.000 1.000
  Y2                   1.140     1.1343     0.0342     0.0102     0.0012
0.440 1.000

Between Level

 Y1       WITH
  Y2                   0.000     0.0064     0.2005     0.1995     0.0398
0.950 0.050

 Means
  Y1                   0.000    -0.0042     0.1164     0.1130     0.0134
0.950 0.050
  Y2                   0.000    -0.0033     0.1163     0.1101     0.0134
0.930 0.070

 Variances
  Y1                   0.500     3.0783     0.2732     0.2887     6.7215
0.000 1.000
  Y2                   0.500     2.9831     0.2576     0.2765     6.2314
0.000 1.000


CORRELATIONS AND MEAN SQUARE ERROR OF THE TRUE FACTOR VALUES AND THE
FACTOR SCORES

                           CORRELATIONS              MEAN SQUARE ERROR
                       Average    Std. Dev.           Average    Std. Dev.
  Y1                    -0.013       0.061              1.807       0.080
  Y2                     0.006       0.065              1.770       0.080


TECHNICAL 1 OUTPUT


     PARAMETER SPECIFICATION FOR WITHIN

```

```
           NU
              Y1            Y2            Y1^1          Y2^1
              ________      ________      ________      ________
                     0             0             0             0


           LAMBDA
              Y1            Y2            Y1^1          Y2^1
              ________      ________      ________      ________
 Y1                  0             0             0             0
 Y2                  0             0             0             0
 Y1^1                0             0             0             0
 Y2^1                0             0             0             0


           THETA
              Y1            Y2            Y1^1          Y2^1
              ________      ________      ________      ________
 Y1                  0
 Y2                  0             0
 Y1^1                0             0             0
 Y2^1                0             0             0             0


           ALPHA
              Y1            Y2            Y1^1          Y2^1
              ________      ________      ________      ________
                     0             0             0             0


           BETA
              Y1            Y2            Y1^1          Y2^1
              ________      ________      ________      ________
 Y1                  0             0             1             2
 Y2                  0             0             3             4
 Y1^1                0             0             0             0
 Y2^1                0             0             0             0


           PSI
              Y1            Y2            Y1^1          Y2^1
              ________      ________      ________      ________
 Y1                  5
 Y2                  6             7
 Y1^1                0             0             0
 Y2^1                0             0             0             0


     PARAMETER SPECIFICATION FOR BETWEEN


           NU
              Y1            Y2
              ________      ________
                     0             0


           LAMBDA
              Y1            Y2
```

```
              ________      ________
 Y1                0             0
 Y2                0             0


           THETA
              Y1            Y2
              ________      ________
 Y1                0
 Y2                0             0


           ALPHA
              Y1            Y2
              ________      ________
                   8             9


           BETA
              Y1            Y2
              ________      ________
 Y1                0             0
 Y2                0             0


           PSI
              Y1            Y2
              ________      ________
 Y1               10
 Y2               11            12


     STARTING VALUES FOR WITHIN


           NU
              Y1            Y2            Y1^1          Y2^1
              ________      ________      ________      ________
                0.000         0.000         0.000         0.000


           LAMBDA
              Y1            Y2            Y1^1          Y2^1
              ________      ________      ________      ________
 Y1             1.000         0.000         0.000         0.000
 Y2             0.000         1.000         0.000         0.000
 Y1^1           0.000         0.000         1.000         0.000
 Y2^1           0.000         0.000         0.000         1.000


           THETA
              Y1            Y2            Y1^1          Y2^1
              ________      ________      ________      ________
 Y1             0.000
 Y2             0.000         0.000
 Y1^1           0.000         0.000         0.000
 Y2^1           0.000         0.000         0.000         0.000


           ALPHA
```

```
              Y1            Y2            Y1^1          Y2^1
           ________      ________      ________      ________
              0.000         0.000         0.000         0.000


           BETA
              Y1            Y2            Y1^1          Y2^1
           ________      ________      ________      ________
 Y1           0.000         0.000         0.600         0.000
 Y2           0.000         0.000         0.000         0.400
 Y1^1         0.000         0.000         0.000         0.000
 Y2^1         0.000         0.000         0.000         0.000


           PSI
              Y1            Y2            Y1^1          Y2^1
           ________      ________      ________      ________
 Y1           1.140
 Y2           0.500         1.140
 Y1^1         0.000         0.000         0.500
 Y2^1         0.000         0.000         0.000         0.500


     STARTING VALUES FOR BETWEEN


           NU
              Y1            Y2
           ________      ________
              0.000         0.000


           LAMBDA
              Y1            Y2
           ________      ________
 Y1           1.000         0.000
 Y2           0.000         1.000


           THETA
              Y1            Y2
           ________      ________
 Y1           0.000
 Y2           0.000         0.000


           ALPHA
              Y1            Y2
           ________      ________
              0.000         0.000


           BETA
              Y1            Y2
           ________      ________
 Y1           0.000         0.000
 Y2           0.000         0.000


           PSI
```

```
                 Y1            Y2
              ________      ________
 Y1             0.500
 Y2             0.000         0.500


     POPULATION VALUES FOR WITHIN


           NU
              Y1            Y2            Y1^1          Y2^1
              ________      ________      ________      ________
                0.000         0.000         0.000         0.000


           LAMBDA
              Y1            Y2            Y1^1          Y2^1
              ________      ________      ________      ________
 Y1             1.000         0.000         0.000         0.000
 Y2             0.000         1.000         0.000         0.000
 Y1^1           0.000         0.000         1.000         0.000
 Y2^1           0.000         0.000         0.000         1.000


           THETA
              Y1            Y2            Y1^1          Y2^1
              ________      ________      ________      ________
 Y1             0.000
 Y2             0.000         0.000
 Y1^1           0.000         0.000         0.000
 Y2^1           0.000         0.000         0.000         0.000


           ALPHA
              Y1            Y2            Y1^1          Y2^1
              ________      ________      ________      ________
                0.000         0.000         0.000         0.000


           BETA
              Y1            Y2            Y1^1          Y2^1
              ________      ________      ________      ________
 Y1             0.000         0.000         0.600         0.000
 Y2             0.000         0.000         0.000         0.400
 Y1^1           0.000         0.000         0.000         0.000
 Y2^1           0.000         0.000         0.000         0.000


           PSI
              Y1            Y2            Y1^1          Y2^1
              ________      ________      ________      ________
 Y1             0.000
 Y2             0.000         0.000
 Y1^1           0.000         0.000         1.000
 Y2^1           0.000         0.000         0.000         1.000


     POPULATION VALUES FOR BETWEEN
```

```
           NU
              Y1            Y2
              ________      ________
                0.000         0.000


           LAMBDA
              P1            P2            P3            Y1            Y2
              ________      ________      ________      ________      ________
 Y1             0.000         0.000         0.000         1.000         0.000
 Y2             0.000         0.000         0.000         0.000         1.000


           THETA
              Y1            Y2
              ________      ________
 Y1             0.000
 Y2             0.000         0.000


           ALPHA
              P1            P2            P3            Y1            Y2
              ________      ________      ________      ________      ________
                0.500         0.000         0.000         0.000         0.000


           BETA
              P1            P2            P3            Y1            Y2
              ________      ________      ________      ________      ________
 P1             0.000         0.000         0.000         0.000         0.000
 P2             0.000         0.000         0.000         0.000         0.000
 P3             0.000         0.000         0.000         0.000         0.000
 Y1             0.000         0.000         0.000         0.000         0.000
 Y2             0.000         0.000         0.000         0.000         0.000


           PSI
              P1            P2            P3            Y1            Y2
              ________      ________      ________      ________      ________
 P1             0.062
 P2             0.000         0.250
 P3             0.000         0.000         0.250
 Y1             0.000         0.000         0.000         3.000
 Y2             0.000         0.000         0.000         0.000         3.000



     PRIORS FOR ALL PARAMETERS            PRIOR MEAN      PRIOR VARIANCE
PRIOR STD. DEV.

     Parameter 1~N(0.000,infinity)           0.0000            infinity
infinity
     Parameter 2~N(0.000,infinity)           0.0000            infinity
infinity
     Parameter 3~N(0.000,infinity)           0.0000            infinity
infinity
```

```
     Parameter 4~N(0.000,infinity)           0.0000            infinity
infinity
     Parameter 5~IW(0.000,-3)              infinity            infinity
infinity
     Parameter 6~IW(0.000,-3)              infinity            infinity
infinity
     Parameter 7~IW(0.000,-3)              infinity            infinity
infinity
     Parameter 8~N(0.000,infinity)           0.0000            infinity
infinity
     Parameter 9~N(0.000,infinity)           0.0000            infinity
infinity
     Parameter 10~IW(0.000,-3)             infinity            infinity
infinity
     Parameter 11~IW(0.000,-3)             infinity            infinity
infinity
     Parameter 12~IW(0.000,-3)             infinity            infinity
infinity


DIAGRAM INFORMATION

  Mplus diagrams are currently not available for multilevel analysis.
  No diagram output was produced.


     Beginning Time:  15:36:43
        Ending Time:  15:44:47
       Elapsed Time:  00:08:04



MUTHEN & MUTHEN
3463 Stoner Ave.
Los Angeles, CA  90066

Tel: (310) 391-9971
Fax: (310) 391-8971
Web: www.StatModel.com
Support: Support@StatModel.com

Copyright (c) 1998-2023 Muthen & Muthen
```

Fitting wrongly directed lag0

```
Mplus VERSION 8.10
MUTHEN & MUTHEN
09/12/2024   2:03 PM

INPUT INSTRUCTIONS

  TITLE:
  dikj
  MONTECARLO:
  NAMES ARE y1-y2 ;
  NOBSERVATIONS = 25000;
  NREPS = 100;
    NCSIZES = 1; CSIZES = 250(100);
    lagged = y1(1) y2(1);
    SEED = 14;
  MODEL POPULATION:

  %WITHIN%

  p1 | y2 ON y1;
  y1^ ON y1^1@0.6;
  y2^ ON y2^1@0.4;
  p2 | y1;
  p3 | y2;


   %BETWEEN%
   [p1*0.5 p2*0 p3*0];
  p1*0.0625 p2*0.25 p3*0.25;


  y1@3;
  y2@3;
  ANALYSIS:
  TYPE IS TWOLEVEL RANDOM;
    estimator=bayes; proc=8;
    BSEED = 23;
    FBITER = 400;
  MODEL:

   %WITHIN%

  y1 ON y2*0.5;
  y1^ ON y1^1*0.6;
   y2^ ON y2^1*0.4;
  y2^ ON y1^1*0;
  y1^ ON y2^1*0;
  y1*1.14;
  y2*1.14;


   %BETWEEN%

  y1 WITH y2*0;
  OUTPUT:
  Standardized;
```

```
*** WARNING in OUTPUT command
  STANDARDIZED option is not available for MONTECARLO.
  Request for STANDARDIZED is ignored.
   1 WARNING(S) FOUND IN THE INPUT INSTRUCTIONS




dikj

SUMMARY OF ANALYSIS

Number of groups                                                 1
Number of observations                                       25000

Number of replications
    Requested                                                  100
    Completed                                                  100
Value of seed                                                   14

Number of dependent variables                                    2
Number of independent variables                                  2
Number of continuous latent variables                            0

Observed dependent variables

  Continuous
   Y1          Y2

Observed independent variables
   Y1^1        Y2^1

Variables with special functions

  Within variables
   Y1^1        Y2^1


Estimator                                                    BAYES
Specifications for Bayesian Estimation
  Point estimate                                            MEDIAN
  Number of Markov chain Monte Carlo (MCMC) chains               2
  Random seed for the first chain                               23
  Starting value information                           UNPERTURBED
  Algorithm used for Markov chain Monte Carlo           GIBBS(PX1)
  Fixed number of iterations                                   400
  K-th iteration used for thinning                               1


SUMMARY OF DATA FOR THE FIRST REPLICATION

     Cluster information

       Size (s)    Number of clusters of Size s

        100           250
```

```
MODEL FIT INFORMATION

Number of Free Parameters                       12

Information Criteria

    Deviance (DIC)

        Mean                                150379.510
        Std Dev                               1202.509
        Number of successful computations      100

             Proportions                   Percentiles
        Expected    Observed         Expected       Observed
           0.990       1.000       147582.113     147629.628
           0.980       0.980       147909.917     147693.418
           0.950       0.930       148401.502     148192.733
           0.900       0.880       148838.374     148655.187
           0.800       0.830       149367.478     149428.622
           0.700       0.710       149748.914     149764.643
           0.500       0.460       150379.510     150257.242
           0.300       0.330       151010.106     151038.964
           0.200       0.220       151391.541     151526.499
           0.100       0.140       151920.645     152089.525
           0.050       0.050       152357.517     152351.992
           0.020       0.000       152849.103     152475.644
           0.010       0.000       153176.907     152530.020

    Estimated Number of Parameters (pD)

        Mean                                   496.706
        Std Dev                                  6.853
        Number of successful computations      100

             Proportions                   Percentiles
        Expected    Observed         Expected       Observed
           0.990       0.970          480.765        479.862
           0.980       0.960          482.633        479.923
           0.950       0.940          485.434        483.372
           0.900       0.910          487.924        486.763
           0.800       0.830          490.939        491.354
           0.700       0.690          493.113        492.783
           0.500       0.530          496.706        496.897
           0.300       0.290          500.300        500.045
           0.200       0.230          502.473        502.693
           0.100       0.100          505.489        505.358
           0.050       0.040          507.978        506.854
           0.020       0.010          510.780        509.195
           0.010       0.000          512.648        510.741



MODEL RESULTS

                              ESTIMATES              S. E.     M. S. E.
95%  % Sig
```

```
                    Population   Average   Std. Dev.   Average          Cover
Coeff

Within Level

 Y1          ON
  Y2                     0.500      0.3850     0.0326     0.0098     0.0143
0.000 1.000

 Y1^         ON
  Y1^1                   0.600      0.5636     0.0071     0.0055     0.0014
0.000 1.000
  Y2^1                   0.000      0.0579     0.0059     0.0066     0.0034
0.000 1.000

 Y2^         ON
  Y2^1                   0.400      0.4505     0.0076     0.0059     0.0026
0.000 1.000
  Y1^1                   0.000      0.0927     0.0080     0.0068     0.0086
0.000 1.000

 Residual Variances
  Y1                     1.140      0.9155     0.0312     0.0083     0.0514
0.000 1.000
  Y2                     1.140      1.4945     0.0445     0.0135     0.1276
0.000 1.000

Between Level

 Y1        WITH
  Y2                     0.000      0.0040     0.1995     0.2004     0.0394
0.950 0.050

 Means
  Y1                     0.000     -0.0037     0.1164     0.1127     0.0134
0.950 0.050
  Y2                     0.000     -0.0019     0.1173     0.1105     0.0136
0.940 0.060

 Variances
  Y1                     0.500      3.0774     0.2726     0.2877     6.7167
0.000 1.000
  Y2                     0.500      2.9823     0.2590     0.2792     6.2280
0.000 1.000


CORRELATIONS AND MEAN SQUARE ERROR OF THE TRUE FACTOR VALUES AND THE
FACTOR SCORES

                         CORRELATIONS               MEAN SQUARE ERROR
                     Average     Std. Dev.          Average     Std. Dev.
  Y1                  -0.013        0.061            1.808        0.079
  Y2                   0.006        0.065            1.764        0.081


TECHNICAL 1 OUTPUT


     PARAMETER SPECIFICATION FOR WITHIN

```

```
           NU
              Y1            Y2            Y1^1          Y2^1
              ________      ________      ________      ________
                     0             0             0             0


           LAMBDA
              Y1            Y2            Y1^1          Y2^1
              ________      ________      ________      ________
 Y1                  0             0             0             0
 Y2                  0             0             0             0
 Y1^1                0             0             0             0
 Y2^1                0             0             0             0


           THETA
              Y1            Y2            Y1^1          Y2^1
              ________      ________      ________      ________
 Y1                  0
 Y2                  0             0
 Y1^1                0             0             0
 Y2^1                0             0             0             0


           ALPHA
              Y1            Y2            Y1^1          Y2^1
              ________      ________      ________      ________
                     0             0             0             0


           BETA
              Y1            Y2            Y1^1          Y2^1
              ________      ________      ________      ________
 Y1                  0             1             2             3
 Y2                  0             0             4             5
 Y1^1                0             0             0             0
 Y2^1                0             0             0             0


           PSI
              Y1            Y2            Y1^1          Y2^1
              ________      ________      ________      ________
 Y1                  6
 Y2                  0             7
 Y1^1                0             0             0
 Y2^1                0             0             0             0


     PARAMETER SPECIFICATION FOR BETWEEN


           NU
              Y1            Y2
              ________      ________
                     0             0


           LAMBDA
              Y1            Y2
```

```
                 ________      ________
 Y1                     0             0
 Y2                     0             0


           THETA
              Y1            Y2
              ________      ________
 Y1                     0
 Y2                     0             0


           ALPHA
              Y1            Y2
              ________      ________
                        8             9


           BETA
              Y1            Y2
              ________      ________
 Y1                     0             0
 Y2                     0             0


           PSI
              Y1            Y2
              ________      ________
 Y1                    10
 Y2                    11            12


     STARTING VALUES FOR WITHIN


           NU
              Y1            Y2            Y1^1          Y2^1
              ________      ________      ________      ________
                0.000         0.000         0.000         0.000


           LAMBDA
              Y1            Y2            Y1^1          Y2^1
              ________      ________      ________      ________
 Y1             1.000         0.000         0.000         0.000
 Y2             0.000         1.000         0.000         0.000
 Y1^1           0.000         0.000         1.000         0.000
 Y2^1           0.000         0.000         0.000         1.000


           THETA
              Y1            Y2            Y1^1          Y2^1
              ________      ________      ________      ________
 Y1             0.000
 Y2             0.000         0.000
 Y1^1           0.000         0.000         0.000
 Y2^1           0.000         0.000         0.000         0.000


           ALPHA
```

Y1	Y2	Y1^1	Y2^1
0.000	0.000	0.000	0.000

BETA

	Y1	Y2	Y1^1	Y2^1
Y1	0.000	0.500	0.600	0.000
Y2	0.000	0.000	0.000	0.400
Y1^1	0.000	0.000	0.000	0.000
Y2^1	0.000	0.000	0.000	0.000

PSI

	Y1	Y2	Y1^1	Y2^1
Y1	1.140			
Y2	0.000	1.140		
Y1^1	0.000	0.000	0.500	
Y2^1	0.000	0.000	0.000	0.500

STARTING VALUES FOR BETWEEN

NU

Y1	Y2
0.000	0.000

LAMBDA

	Y1	Y2
Y1	1.000	0.000
Y2	0.000	1.000

THETA

	Y1	Y2
Y1	0.000	
Y2	0.000	0.000

ALPHA

Y1	Y2
0.000	0.000

BETA

	Y1	Y2
Y1	0.000	0.000
Y2	0.000	0.000

PSI

```
              Y1            Y2
           ________      ________
 Y1           0.500
 Y2           0.000         0.500


     POPULATION VALUES FOR WITHIN


           NU
              Y1            Y2            Y1^1          Y2^1
           ________      ________      ________      ________
              0.000         0.000         0.000         0.000


           LAMBDA
              Y1            Y2            Y1^1          Y2^1
           ________      ________      ________      ________
 Y1           1.000         0.000         0.000         0.000
 Y2           0.000         1.000         0.000         0.000
 Y1^1         0.000         0.000         1.000         0.000
 Y2^1         0.000         0.000         0.000         1.000


           THETA
              Y1            Y2            Y1^1          Y2^1
           ________      ________      ________      ________
 Y1           0.000
 Y2           0.000         0.000
 Y1^1         0.000         0.000         0.000
 Y2^1         0.000         0.000         0.000         0.000


           ALPHA
              Y1            Y2            Y1^1          Y2^1
           ________      ________      ________      ________
              0.000         0.000         0.000         0.000


           BETA
              Y1            Y2            Y1^1          Y2^1
           ________      ________      ________      ________
 Y1           0.000         0.000         0.600         0.000
 Y2           0.000         0.000         0.000         0.400
 Y1^1         0.000         0.000         0.000         0.000
 Y2^1         0.000         0.000         0.000         0.000


           PSI
              Y1            Y2            Y1^1          Y2^1
           ________      ________      ________      ________
 Y1           0.000
 Y2           0.000         0.000
 Y1^1         0.000         0.000         1.000
 Y2^1         0.000         0.000         0.000         1.000


     POPULATION VALUES FOR BETWEEN
```

```
           NU
              Y1            Y2
              ________      ________
                0.000         0.000


           LAMBDA
              P1            P2            P3            Y1            Y2
              ________      ________      ________      ________      ________
 ________
 Y1           0.000         0.000         0.000         1.000         0.000
 Y2           0.000         0.000         0.000         0.000         1.000


           THETA
              Y1            Y2
              ________      ________
 Y1             0.000
 Y2             0.000         0.000


           ALPHA
              P1            P2            P3            Y1            Y2
              ________      ________      ________      ________      ________
 ________
                0.500         0.000         0.000         0.000         0.000


           BETA
              P1            P2            P3            Y1            Y2
              ________      ________      ________      ________      ________
 ________
 P1           0.000         0.000         0.000         0.000         0.000
 P2           0.000         0.000         0.000         0.000         0.000
 P3           0.000         0.000         0.000         0.000         0.000
 Y1           0.000         0.000         0.000         0.000         0.000
 Y2           0.000         0.000         0.000         0.000         0.000


           PSI
              P1            P2            P3            Y1            Y2
              ________      ________      ________      ________      ________
 ________
 P1             0.062
 P2             0.000         0.250
 P3             0.000         0.000         0.250
 Y1             0.000         0.000         0.000         3.000
 Y2           0.000         0.000         0.000         0.000         3.000



     PRIORS FOR ALL PARAMETERS            PRIOR MEAN      PRIOR VARIANCE
PRIOR STD. DEV.

     Parameter 1~N(0.000,infinity)          0.0000            infinity
infinity
     Parameter 2~N(0.000,infinity)          0.0000            infinity
infinity
     Parameter 3~N(0.000,infinity)          0.0000            infinity
infinity
```

```
     Parameter 4~N(0.000,infinity)            0.0000            infinity
infinity
     Parameter 5~N(0.000,infinity)            0.0000            infinity
infinity
     Parameter 6~IG(-1.000,0.000)           infinity            infinity
infinity
     Parameter 7~IG(-1.000,0.000)           infinity            infinity
infinity
     Parameter 8~N(0.000,infinity)            0.0000            infinity
infinity
     Parameter 9~N(0.000,infinity)            0.0000            infinity
infinity
     Parameter 10~IW(0.000,-3)              infinity            infinity
infinity
     Parameter 11~IW(0.000,-3)              infinity            infinity
infinity
     Parameter 12~IW(0.000,-3)              infinity            infinity
infinity
```

DIAGRAM INFORMATION

Mplus diagrams are currently not available for multilevel analysis.
No diagram output was produced.

```
     Beginning Time:  14:03:37
        Ending Time:  14:12:30
       Elapsed Time:  00:08:53
```

MUTHEN & MUTHEN
3463 Stoner Ave.
Los Angeles, CA 90066

Tel: (310) 391-9971
Fax: (310) 391-8971
Web: www.StatModel.com
Support: Support@StatModel.com

Copyright (c) 1998-2023 Muthen & Muthen

References

Debbané, M., Eliez, S., Badoud, D., Conus, P., Flückiger, R., & Schultze-Lutter, F. (2015). Developing Psychosis and Its Risk States Through the Lens of Schizotypy. *Schizophrenia Bulletin*, *41*(suppl_2), S396–S407. doi: 10.1093/schbul/sbu176

Fusar-Poli, P., Borgwardt, S., Bechdolf, A., Addington, J., Riecher-Rössler, A., Schultze-Lutter, F., … Yung, A. (2013). The Psychosis High-Risk State: A Comprehensive State-of-the-Art Review. *JAMA Psychiatry*, *70*(1), 107. doi: 10.1001/jamapsychiatry.2013.269

Fusar-Poli, P., Borgwardt, S., & Valmaggia, L. (2008). Heterogeneity in the assessment of the at-risk mental state for psychosis. *Psychiatric Services (Washington, D.C.)*, *59*(7), 813. doi: 10.1176/ps.2008.59.7.813

Fusar-Poli, P., Cappucciati, M., Borgwardt, S., Woods, S. W., Addington, J., Nelson, B., … McGuire, P. K. (2016). Heterogeneity of Psychosis Risk Within Individuals at Clinical High Risk: A Meta-analytical Stratification. *JAMA Psychiatry*, *73*(2), 113. doi: 10.1001/jamapsychiatry.2015.2324

Fusar-Poli, P., Raballo, A., & Parnas, J. (2017). What Is an Attenuated Psychotic Symptom? On the Importance of the Context. *Schizophrenia Bulletin*, *43*(4), 687–692. doi: 10.1093/schbul/sbw182

Fux, L., Walger, P., Schimmelmann, B. G., & Schultze-Lutter, F. (2013). The Schizophrenia Proneness Instrument, Child and Youth version (SPI-CY): Practicability and discriminative validity. *Schizophrenia Research*, *146*(1–3), 69–78. doi: 10.1016/j.schres.2013.02.014

Jones, P. B. (2002). Risk factors for schizophrenia in childhood and youth. In H. Häfner (Ed.), *Risk and Protective Factors in Schizophrenia* (pp. 141–162). Heidelberg: Steinkopff. doi: 10.1007/978-3-642-57516-7_13

Keshavan, M. S., DeLisi, L. E., & Seidman, L. J. (2011). Early and broadly defined psychosis risk mental states. *Schizophrenia Research*, *126*(1–3), 1–10. doi: 10.1016/j.schres.2010.10.006

Klosterkotter, J., Schultze-Lutter, F., Bechdolf, A., & Ruhrmann, S. (2011). Prediction and prevention of schizophrenia: What has been achieved and where to go next? *World Psychiatry*, *10*(3), 165–174. doi: 10.1002/j.2051-5545.2011.tb00044.x

McGlashan, T., Walsh, B., & Woods, S. (2010). *The psychosis-risk syndrome: Handbook for diagnosis and follow-up*. Oxford University Press.

Michel, C., Lerch, S., Büetiger, J. R., Flückiger, R., Cavelti, M., Koenig, J., … Kindler, J. (2022). An ecological momentary assessment study of age effects on perceptive and non-perceptive clinical high-risk symptoms of psychosis. *European Child & Adolescent Psychiatry*. doi: 10.1007/s00787-022-02003-9

Parnas, J., & Carter, J. W. (2002). High-risk studies and neurodevelopmental hypothesis. In H. Häfner (Ed.), *Risk and Protective Factors in Schizophrenia* (pp. 71–82). Heidelberg: Steinkopff. doi: 10.1007/978-3-642-57516-7_6

Phillips, L. J., Yung, A. R., & McGorry, P. D. (2000). Identification of young people at risk of psychosis: Validation of Personal Assessment and Crisis Evaluation Clinic intake criteria. *The Australian and New Zealand Journal of Psychiatry*, *34 Suppl*, S164-169. doi: 10.1080/000486700239

Phillips, Lisa J., Yung, A. R., & McGorry, P. D. (2000). Identification of Young People at Risk of Psychosis: Validation of Personal Assessment and Crisis Evaluation Clinic Intake Criteria. *Australian & New Zealand Journal of Psychiatry*, *34*(1_suppl), A164–A169. doi: 10.1177/000486740003401S25

Reininghaus, U., Gayer-Anderson, C., Valmaggia, L., Kempton, M. J., Calem, M., Onyejiaka, A., … Morgan, C. (2016). Psychological processes underlying the association between

childhood trauma and psychosis in daily life: An experience sampling study. *Psychological Medicine*, *46*(13), 2799–2813. doi: 10.1017/S003329171600146X

Ruhrmann, S., Schultze-Lutter, F., Salokangas, R. K. R., Heinimaa, M., Linszen, D., Dingemans, P., … Klosterkötter, J. (2010). Prediction of psychosis in adolescents and young adults at high risk: Results from the prospective European prediction of psychosis study. *Archives of General Psychiatry*, *67*(3), 241–251. doi: 10.1001/archgenpsychiatry.2009.206

Schultze-Lutter, F. (2009). Subjective Symptoms of Schizophrenia in Research and the Clinic: The Basic Symptom Concept. *Schizophrenia Bulletin*, *35*(1), 5–8. doi: 10.1093/schbul/sbn139

Schultze-Lutter, F., Michel, C., Schmidt, S. J., Schimmelmann, B. G., Maric, N. P., Salokangas, R. K. R., … Klosterkötter, J. (2015). EPA guidance on the early detection of clinical high risk states of psychoses. *European Psychiatry*, *30*(3), 405–416. doi: 10.1016/j.eurpsy.2015.01.010

Schultze-Lutter, F., Addington, J., Ruhrmann, S., & Klosterkötter, J. (2007). Schizophrenia proneness instrument, adult version (SPI-A). *Rome: Giovanni Fioriti*.

Schultze-Lutter, F., Debbané, M., Theodoridou, A., Wood, S. J., Raballo, A., Michel, C., … Uhlhaas, P. J. (2016). Revisiting the Basic Symptom Concept: Toward Translating Risk Symptoms for Psychosis into Neurobiological Targets. *Frontiers in Psychiatry*, *7*. doi: 10.3389/fpsyt.2016.00009

Schultze-Lutter, F., Ruhrmann, S., Fusar-Poli, P., Bechdolf, A., G. Schimmelmann, B., & Klosterkotter, J. (2012). Basic Symptoms and the Prediction of First-Episode Psychosis. *Current Pharmaceutical Design*, *18*(4), 351–357. doi: 10.2174/138161212799316064

Shrivastava, A., McGorry, P., Tsuang, M., Woods, S., Cornblatt, B., Corcoran, C., & Carpenter, W. (2011). ‘Attenuated psychotic symptoms syndrome’ as a risk syndrome

of psychosis, diagnosis in DSM-V: The debate. *Indian Journal of Psychiatry*, *53*(1), 57. doi: 10.4103/0019-5545.75560

Yung, A. R., Stanford, C., Cosgrave, E., Killackey, E., Phillips, L., Nelson, B., & McGorry, P. D. (2006). Testing the Ultra High Risk (prodromal) criteria for the prediction of psychosis in a clinical sample of young people. *Schizophrenia Research*, *84*(1), 57–66. doi: 10.1016/j.schres.2006.03.014
